# Sugar-Based Surfactants: Effects of Structural Features on the Physicochemical Properties of Sugar Esters and Their Comparison to Commercial Octyl Glycosides

**DOI:** 10.3390/molecules29102338

**Published:** 2024-05-16

**Authors:** Huiling Lu, Gwladys Pourceau, Benoit Briou, Anne Wadouachi, Théophile Gaudin, Isabelle Pezron, Audrey Drelich

**Affiliations:** 1 Université de Technologie de Compiègne, ESCOM, TIMR (Transformations Intégrées de la Matière Renouvelable), Centre de Recherche Royallieu—CS 60 319, 60203 Compiègne Cedex, France; lhl@ujs.edu.cn (H.L.); isabelle.pezron@utc.fr (I.P.); 2 Laboratoire de Glycochimie, et des Agroressources d’Amiens (LG2A), UR 7378—Institut de Chimie de Picardie, Université de Picardie Jules Verne, 33 rue Saint Leu, 80039 Amiens Cedex, France; b.briou@orpiainnovation.com (B.B.); anne.wadouachi@u-picardie.fr (A.W.)

**Keywords:** sugar esters, carbohydrate-based surfactants, glycolipids, physicochemical parameters

## Abstract

Two series of sugar esters with alkyl chain lengths varying from 5 to 12 carbon atoms, and with a head group consisting of glucose or galactose moieties, were synthesized. Equilibrium surface tension isotherms were measured, yielding critical micellar concentration (CMC) surface tensions at CMC (γcmc) and minimum areas at the air–water interface (Amin). In addition, Krafft temperatures (Tks) were measured to characterize the ability of molecules to dissolve in water, which is essential in numerous applications. As a comparison to widely used commercial sugar-based surfactants, those measurements were also carried out for four octyl d-glycosides. Impacts of the linkages between polar and lipophilic moieties, alkyl chain lengths, and the nature of the sugar head group on the measured properties were highlighted. Higher Tk and, thus, lower dissolution ability, were found for methyl 6-*O*-acyl-d-glucopyranosides. CMC and γcmc decreased with the alkyl chain lengths in both cases, but Amin did not appear to be influenced. Both γcmc and Amin appeared independent of the ester group orientation. Notably, alkyl (methyl α-d-glucopyranosid)uronates were found to result in noticeably lower CMC, possibly due to a closer distance between the carbonyl function and the head group.

## 1. Introduction

Glycolipids are often considered safe alternatives to petroleum-based surfactants due to their renewable origin, high biodegradability, and low toxicity. Their surface-active properties make them highly interesting in a wide range of fields such as food, cosmetics, pharmaceuticals, and detergent industries [1,2,3,4,5]. These amphiphilic molecules comprise a saccharidic polar head group linked to a lipophilic chain. Depending on the linkage, its orientation and position, the length and degree of saturation of the alkyl chain, the sugar residue and its size, etc., a wide variety of sugar-based amphiphiles can be designed. Despite the fact that many studies have focused on relationships between their chemical structures and surface properties, important gaps remain, owing to the structural versatility of sugar-based surfactants [6,7,8,9]. Filling those gaps (regarding the knowledge of structure–property relationships) will help identify and anticipate trends, with the final aim of designing green bio-based surfactants for specific applications.

**Figure 1:**
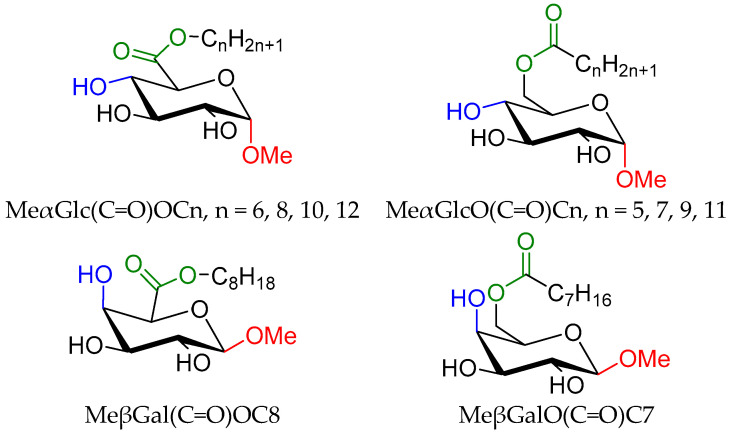
Structures of 1-*O*-methyl alkyl-d-glycuronates [29] (**left**) and methyl 6-*O*-acyl-d-glycopyranosides (**right**). Each molecule is abbreviated with the code annotated below.

The structures of sugar-based surfactants are diverse (including glyco-amides, glyco-esters, glycosides, and other derivatives), and their properties also widely vary. For example, in previous work [10], we synthesized various glyco-amides, bearing an octyl chain, which differed by the position and the orientation of the amide linkage on the sugar head-group (Su-NHCO-R or Su-CONH-R), and measured significant differences for those molecules in physicochemical properties, such as solubility or CMC, as well as in cytotoxic effects. In particular, esterase enzymes found widely in nature can break down sugar esters, leading to high biodegradability for this specific surfactant family, and making it relevant whenever eco-compatibility is a concern [11]. Another advantage of sugar esters is the general ease of their synthesis, as the ester linkage can be obtained by simple chemical [12,13,14] or enzymatic routes [15,16,17,18]. However, their ability to dissolve in water appears to be lower than that of commercial alkyl glycosides, a difference observed across a diverse range of sugar residues combined with ester linkages (alkyl uronates, alkyl aldonates, acyl oses, acyl itols, etc.) [19,20,21,22,23,24]. Moreover, in most studies, mixtures of α and β isomers with an undetermined ratio amount are reported, even though this structural parameter influences dissolution and micellization [25,26,27,28]. As reported by Brown et al. [25], the solubility of octyl-β-d-glucoside (C8βGlc) is about seven times larger than that of octyl-α-d-glucoside (C8αGlc), due to the latter’s more stable crystal structure. Consistently, glucosides with α-linkages are generally believed to have higher Krafft temperatures (Tks) than those with β-linkages [26]. Thus, it is essential to know exactly the α/β ratio or to protect the anomeric position to establish structure/function relationships. It is also necessary to keep in mind that the replacement of the anomeric -OH function with other groups should cause a decreased solubility of the corresponding esters at 25 °C [20]. In 2016, we reported on the synthesis and physicochemical behavior of one family of sugar esters with well-defined anomeric configurations, namely 1-*O*-methyl alkyl uronates (MeαGlc, MeβGal, and MeαMan derivatives) (Figure 1 left) [29]. This study revealed that, similar to corresponding alkyl polyglycosides or common polyoxyethylene nonionic surfactants, our newly synthesized sugar esters were able to significantly reduce the surface tensions of aqueous solutions, at low concentrations. Nevertheless, their solubility remained limited.

Thus, we synthesized and characterized close, but distinct sugar esters with well-defined anomeric positions, derived from MeαGlc and MeβGal: methyl 6-*O*-acyl-d-glycopyranosides (Figure 1 right). Their physicochemical properties were compared to the corresponding 1-*O*-methyl alkyl uronates that we previously described. Thanks to the homology of both families (the same nature and position of the linkage, and the same anomeric configuration), the effects of other structural parameters, including the chain length (hexyl to dodecyl derivatives), the orientation of the ester groups, as well as the sugar head, can be directly compared.

**Figure 2:**
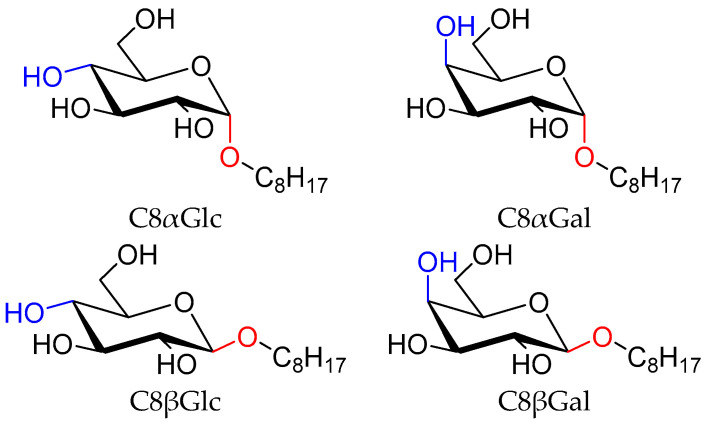
Structures of octyl-α/β-d-glucosides (**left**) and octyl-α/β-d-galactosides (**right**). Each molecule is abbreviated with the code annotated below.

Commercial alkyl α/β-glycosides have been, by far, the most extensively investigated sugar-based surfactants, making them relevant comparison models. Indeed, their structures are a straightforward combination of a typical sugar residue, such as glucose, with an alkyl chain, i.e., a prototypical surfactant that can be imagined with sugar as a polar head. Nevertheless, confidence in the available knowledge of their physicochemical behavior is limited by significant inconsistency and discrepancy for the same structure sometimes apparent within a large amount of published physicochemical measurements, as we highlighted previously [30]. For example, in 1997, Sakya et al. showed—for the first time—the phase diagram of octyl-β-d-galactoside (C8βGal), from which a Tk of 42 °C was deduced by Hato et al. [31]. In parallel, several researchers have measured the surface tension of C8βGal directly at 25 °C, assuming that the surfactant properly dissolves in water at this temperature [32,33]. To clarify the observed discrepancies in the literature regarding the structure–property relationships for alkyl glycosides, in the present study, properties for two pairs of α/β anomers, i.e., C8αGlc/C8βGlc and C8αGal/C8βGal, have also been investigated (Figure 2).

## 2. Results and Discussion

### 2.1. Synthesis

**Scheme 1:**
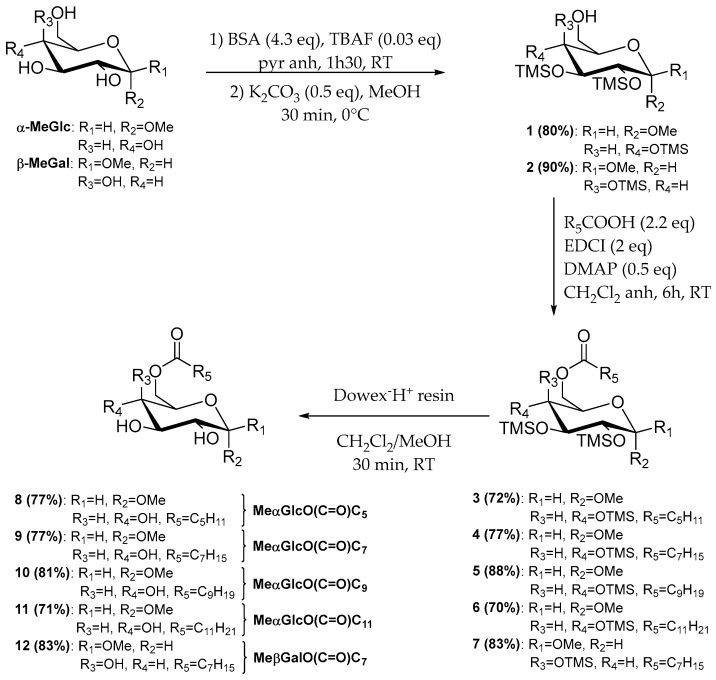
Synthesis of methyl 6-*O*-acyl-d-glycopyranosides.

Two families of sugar esters, alkyl (methyl d-glycopyranosid)uronates, and methyl 6-*O*-acyl-d-glycopyranosides, were synthesized. The uronates family was obtained using the two-step procedure that we previously described [29]. For the methyl 6-*O*-acyl-d-glycopyranosides, a known strategy ensuring regioselectivity on OH-6 [34] was successfully adapted and applied to methyl d-glycosides (Scheme 1). Firstly, α-methyl d-glucoside was per-silylated by treating with *N*,*O*-bis(trimethylsilyl)acetamide (BSA) and catalytic tetrabutylammonium fluoride (TBAF), and the more labile primary silyl ether was deprotected in a one-pot procedure during the work-up by the addition of K_2_CO_3_ to generate methyl 2,3,4-tri-*O*-trimethylsilyl-α-d-glucopyranoside **1** with an 80% yield. Methyl 2,3,4-tri-*O*-trimethylsilyl-β-d-galactopyranoside **2** was obtained with a 90% yield following the same protocol, starting from β-methyl d-galactoside. The TMS-protected derivative was then coupled to the fatty carboxylic acid by esterification mediated by 1-ethyl-3-(3-dimethylaminopropyl)carbodiimide (EDCI) and 4-(dimethylamino)pyridine (DMAP), leading to methyl 6-*O*-acyl-2,3,4-tri-*O*-trimethylsilyl-d-glycopyranosides **3**–**7** with good to excellent yields (70–88% yield, depending on the alkyl chain and the sugar head group). After quantitative deprotection of the TMS groups using Dowex-H^+^ resin and purification on normal phase chromatography, methyl 6-*O*-acyl-α-d-glucopyranosides with C6, C8, C10 and C12 alkyl chains **8**–**11** and methyl 6-*O*-octanoyl-β-d-galactopyranoside **12** were obtained at the 2g-scale with good to excellent yields (71–83%). The high purity of each intermediate and final compound was confirmed by ^1^H and ^13^C NMR (reported in the Supplementary Materials) and HRMS.

For the sake of brevity, from now on, methyl 6-*O*-acyl-α-d-glycopyranosides will be named MeαGlcO(C=O)Cn (n = 5, 7, 9, 11) and MeβGalO(C=O)C7, respectively, whereas alkyl (methyl d-glycopyranosid)uronates will be called MeαGlc(C=O)OCn (n = 6, 8, 10, 12) and MeβGal(C=O)OC8.

### 2.2. Solubility

**Figure 3:**
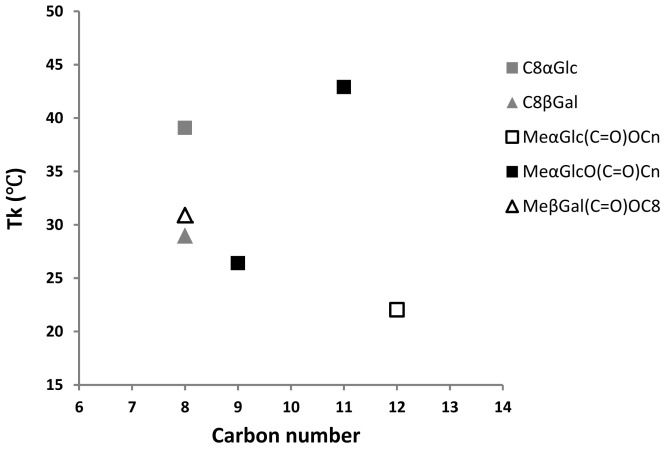
Tk as a function of alkyl chain length for studied molecules.

Apart from the alkyl chain length, a cornerstone surfactant characteristic, it is generally believed that for sugar-based surfactants in particular, variations in physicochemical properties, including solubility, are often caused by differences in the molecular shape and intra/intermolecular hydrogen bonding [35]. Tk represents the temperature above which micelles can form. Below Tk, surfactants in hydrated solid form coexist with monomers at their solubility limit, which is visually determined at the transparency limit of the observed solutions. For example, for alkyl glucosides, the Tks of α-anomers are higher than those of the β-anomers and the β-anomers are much more soluble in water than the α-anomers [3]. Table 1 presents the results obtained from both the observation and DSC analysis. In Figure 3, Tk is plotted vs. the alkyl chain length for the various studied surfactant families. The results show that, although important, the alkyl chain length is far from the only relevant factor in Tk values for sugar-based surfactants. Indeed, the configuration at the anomeric center, as well as the relative orientation of one -OH of the polar head group can also have an important impact.

For commercial alkyl glucosides, the Tk decreases in the following sequence: C8αGlc > C8βGal >> C8βGlc ≈ C8αGal. All of them possess appreciable solubility limits above their Tks. Many experimental investigations have reported that C8βGlc is very soluble in water and, therefore, it has a Tk below room temperature, as is also evidenced by our results. The obtained Tk for C8αGlc is around 40 °C in accordance with previously published values [31,36]. However, as determined by the same experimental protocol, the Tk for C8βGal is about 29 °C, much lower than 42 °C derived from the phase diagram reported by Sakya et al. [37] A possible explanation is that Sakya et al. synthesized and purified C8βGal by themselves, whereas we carried our measurements on commercially purchased C8βGal. Interestingly, in contrast with C8αGlc, C8βGal displays a marked solubility at room temperature even if it is below its Tk. A solution containing 20 mM of C8βGal is nearly transparent at room temperature, with only a very low turbidity. For this reason, we assume that many researchers have been able to investigate their aqueous solutions at 25 °C, as mentioned in the introduction. In addition, when cooling the previously heated solutions with the same molecular concentration of 50 mM, we noted that C8αGlc precipitated much faster than C8βGal. The precipitation of C8αGlc took place within an hour, whereas no change was observed over one month for the C8βGal solution. This may, to some extent, be explained by a more favorable crystal structure for C8αGlc than C8βGal. With regard to C8αGal, which is presented in commercial samples as a highly viscous liquid, we expect it to have the weakest crystal structure and a very low Tk value. However, our experimental methods cannot give its exact Tk. Accordingly, one important conclusion can be drawn from our results: for the given type of octyl glycosides, a combination of “axial 4-OH and axial 1-alkyl chain” or “equatorial 4-OH and equatorial 1-alkyl chain”, causes lower Tk values. While a combination like “axial 4-OH and equatorial 1-alkyl chain” or “equatorial 4-OH and axial 1-alkyl chain” can generate higher Tk values. This observation suggests that a different orientation for 4-OH and 1-alkyl chain promotes crystal packing.

After studying commercially available and well-known alkyl glycosides, we focused on the synthesized sugar esters. The observed dissolution phenomena are harder to interpret compared to commercial molecules. This can be a result of more structural contributions such as axial/equatorial 4-OH and 1-OMe, as well as two possible ester orientations. In the case of MeαGlc-derived esters, the solubility decreases, as expected, with the increase of the alkyl chain length. All molecules demonstrated the expected behavior of a lower solubility above their Tk, compared to commercial C8αGlc and C8βGlc. Moreover, both octyl glucuronate (MeαGlc(C=O)OC8) and octanoyl glycoside (MeαGlcO(C=O)C7) derivatives showed lower Tk than C8αGlc. A possible interpretation is that compared to the ether linker, the ester linker creates more disbalance toward hydrogen acceptor groups, which would not find hydrogen donor partners in the crystal structure but would find them in water, increasing aqueous solubility. As highlighted in Table 1, the alkyl (methyl α-d-glycopyranosid)uronates tend to show lower Tk values than their homologous methyl 6-*O*-acyl-α-d-glycopyranosides. This result is also in accordance with how the molecule presents when its pure form is exposed to some limited atmospheric water. Indeed, we could expect that a molecule showing up as a liquid or paste will likely have a lower Tk compared to a molecule presenting as a crystal in those circumstances. For instance, MeαGlc(C=O)OC8 presents as a semi-solid grease while MeαGlcO(C=O)C7 is a solid powder. No obvious structural interpretation arises regarding this difference. One could speculate that the C=O group in methyl 6-*O*-acyl-α-d-glycopyranosides is more available to form tight hydrogen bonding networks compared to alkyl (methyl α-d-glycopyranosid)uronates, where it lies closer to the hydrogen donors on the polar head.

For methyl 6-*O*-acyl-α-D-glucopyranosides, Tk is found to increase with the alkyl chain length, as is the case for alkyl α-glucopyranosides [38]. Moreover, according to Otto et al. [20], the 6-*O*-octanoyl-β-glucose, above its Tk, has a solubility limit of 25 mg/mL (>80 mM), which is much higher than that of our MeαGlcO(C=O)C7 (<10 mM). This indicates that replacing the anomeric -OH of the glucose with a methyl ether group increases hydrophobicity, making MeαGlcO(C=O)C7 less soluble in water.

In the case of MeβGal-derived esters, both methyl octyl galacturonate (MeβGal(C=O)OC8) and methyl 6-*O*-octanoyl galactoside (MeβGalO(C=O)C7) derivatives have high solubility limits above their Tks, not in line with MeαGlc-derived esters.

**Figure 4:**
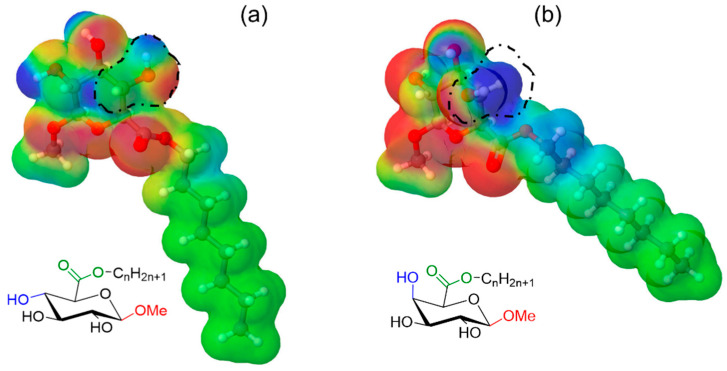
A 3D representation of (**a**) MeβGlcO(C=O)C8 and (**b**) MeβGalO(C=O)C8.

The measured Tk for MeβGalO(C=O)C7 is a little higher than that of C8βGal. Note that contrary to MeαGlc-derived C7/C8 esters, a carbonyl group closer to the sugar residue is associated with a lower ability to dissolve the molecule at room temperature. One hypothesis is that the polar alcohol group in axial orientation leads to a more compact polar head for Gal surfactants, shielding some polar surface area from contact with the solvent. To have a molecular perspective on this hypothesis, we carried out a comparison with the MeβGlcO(C=O)C8 equivalent. Electrostatic potential maps were obtained with Jmol software after a semi-empirical geometry optimization [39]. The results are displayed in Figure 4.

It can be seen that the polar head has a more compact shape; the axial OH is more restricted and has less potential for solvent exposure, supporting a slightly higher hydrophobicity.

### 2.3. Surface Activity

The aqueous tensiometric properties of all the studied molecules are listed in Table 2. CMCs are determined from the inflections in the plots of equilibrium surface tension against the logarithm of the concentration. For the interested reader, the Gibbs-free energy of micellization, ΔG_mic_, the Gibbs-free energy of adsorption, ΔG_ads_, and the efficiency of surfactant adsorption pC_20_ were calculated based on their definitions [40], as presented in Table 2.

Firstly, in the case of octyl d-glycosides, our measured CMC for C8βGlc, 21.2 mM, was consistent with the published values (18–26 mM), despite the numerous experimental methods used by the different authors [41,42,43,44,45]. However, there were discrepancies in the literature for C8αGlc, C8αGal, and C8βGal regarding both CMC and γcmc. Matsumura et al. [32] measured the static surface tension of C8αGlc and C8βGal by the Wilhelmy plate method at 25 °C. The authors have reported, respectively, CMC values of 12 mM and 16 mM. Both showed a γcmc of 30.5 mN/m. We measured the CMC values of 15.5 mM and 20 mM, respectively, higher than the published ones, as well as γcmc 30.7 mN/m and 28.5 mN/m, respectively, indicating a higher effectiveness of surface tension reduction for C8βGal. We questioned their results as the measurements were performed below their Tk (39 °C and 29 °C, respectively, cf. Table 1). Schmidt-Lassen et al. [33] measured the CMC values for C8βGal and C8αGal by using isothermal titration calorimetry (ITC) and found that CMC values at 25 °C were 31.7 ± 0.7 mM for C8βGal and 30.2 ± 0.4 mM for C8αGal, showing no evident anomeric effect on the CMC. In contrast, we found CMCs of 20 mM for C8βGal and 51.7 mM for C8αGal, respectively, indicating that the anomeric orientation has a significant impact on the CMC here. Because the limited aqueous solubility of C8βGal was mentioned, again, we question the results published in their study.

After comparing our measurements with published ones, we focused on the structural interpretations arising from our own measurements. The order of their CMC for the 4 octyl glycosides was: C8αGal > C8βGlc ~ C8βGal > C8αGlc. Here, even at a constant alkyl chain length, a lower CMC correlates with a higher Tk. Apart from the alkyl chain length, the most obvious factor, it is known that polar head hydrophilicity can also significantly influence CMC, typically increasing it for more hydrophilic polar heads [6]. With all else equal, a higher CMC leads to a decrease in Tk due to the thermodynamic relationship between the two. Thus, the observed Tk could also reflect polar head hydrophilicity. As for γcmc, it was found that octyl d-galactosides can more effectively reduce the surface tension of pure water than octyl glucosides. Our Amin measurements suggest that the anomeric effect is more evident for C8α- and C8βGlc than for C8α- and C8βGal. In addition, the latter shows a greater Amin than the former, in agreement with Razafindralambo et al. [14]. It was said that an axial -OH at the 4-position (Gal) gives a larger minimum area occupied per molecule at the water surface. However, as the Amin depends strongly on the way of plotting the **γ** vs. log C curves, different authors have demonstrated different Amin values. Matsumura et al. [32] reported a range of 47–54 Å^2^/molecule for C8–C12 alkyl d-glucosides and galactosides. Their study did not show any great difference between their α- and β-anomers but a slightly larger Amin for C8- and C10 βGal than for the C8- and C10αGlc. Kjellin et al. [23] showed an Amin of 38 Å^2^/molecule for C8βGlc whereas an Amin of 42 Å^2^/molecule was given for C8βGlc by Shinoda et al. [46]. Therefore, our results seem to be in good agreement with the literature.

**Figure 5:**
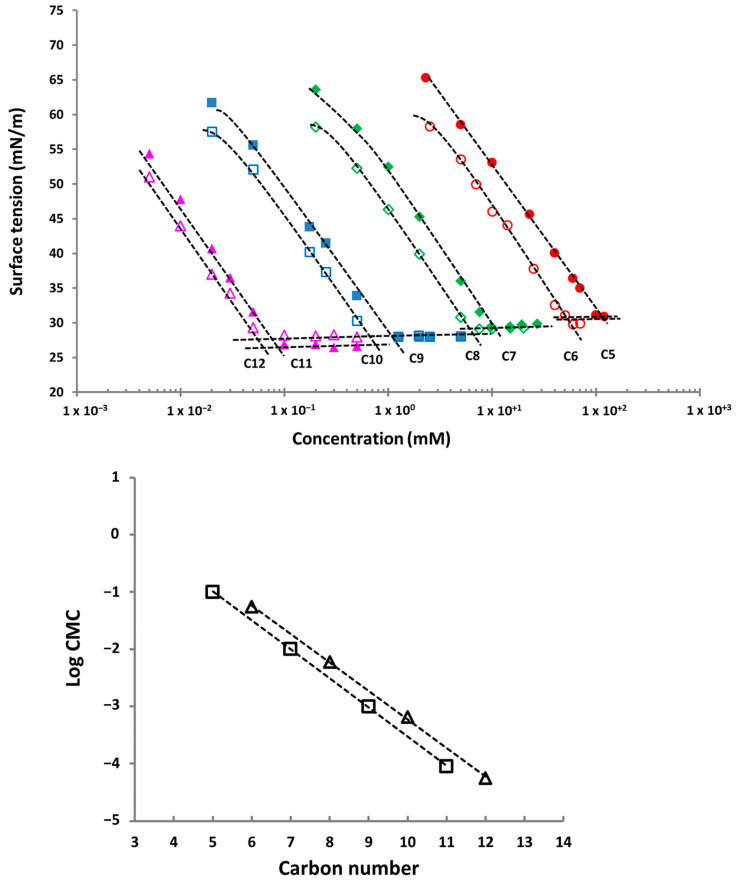
Surface tension as a function of concentration for methyl alkyl α-d-glucuronates at 25 °C (empty markers, MeαGlc(C=O)OCn, with n being an even number) and methyl 6-*O*-acyl-α-d-glucosides (filled markers, MeαGlcO(C=O)Cn, with n being an odd number) at 25 °C (C5 and C7), 30 °C (C9) and 50 °C (C11) (**top**) and the variation of the log CMC with the carbon numbers of the alkyl chain for alkyl methyl α-d-glucuronates (MeαGlc(C=O)OCn, △) and methyl 6-*O*-acyl-α-d-glucosides (MeαGlcO(C=O)Cn, □) (**bottom**). CMC is expressed in mol/L.

The influence of the head saccharidic group of our new surfactants on the surface activity can also be analyzed. Figure 5 (top) represents the surface tension vs. log concentration plots for both families of MeαGlc-derived esters. Firstly, whatever the orientation of the ester group (whatever the family studied), the esters of glucose can efficiently reduce the surface tension of pure water with the obtained low γcmc values of 26–32 mN/m. It seems that the ester group orientation does not impact γcmc much. In general, it can be seen that γcmc slightly decreases with the alkyl chain length. Those results are in line with the identified trends [6] where γcmc appears to decrease asymptotically with the alkyl chain length. The suggested explanation is that as the alkyl chain length increases, alkyl groups get closer and closer to completely covering the surface, making it exhibit an alkane-like γcmc. The only exception comes with MeαGlc(C=O)OC12, which is very close to that of MeαGlc(C=O)OC10, very likely because it is challenging to measure a true γcmc for MeαGlc(C=O)OC12 as it precipitates easily. A similar phenomenon was observed by Otto et al. [20], who reported a higher γcmc for 6-*O*-decanoyl-β-d-glucopyranose (C10) than for 6-*O*-octanoyl-β-d-glucopyranose (C8).

A common conclusion in the literature is that the alkyl chain length is not an important factor in determining the Amin of n-alkyl glucosides at the air–water interface [3]. This observation can probably be extrapolated to sugar esters. Nevertheless, even though Amin is mainly decided by the hydrophilic group [47], the alkyl chain flexibility can contribute to whether molecules loosely or compactly pack at the interface [48]. It is reasonable to assume that given the higher number of rotational degrees of freedom, a longer saturated alkyl chain is more flexible, and based on this argument, we hypothesize that surfactants with longer alkyl chains at a constant polar head can exhibit the Amin increase. Our measurements are consistent with such a hypothesis, with a slight increase of Amin with alkyl chain lengths for both families. A similar trend was mentioned in the literature for other surfactant families, such as bolaform quaternary ammonium surfactants for which the Amin increases with the increase in the spacer carbon number (n < 10) [49]. Nevertheless, it must be noted that for some polyoxyethylene alkyl ethers, Amin decreases with an increasing carbon number but increases with the oxyethylene unit length [47,50,51]. Given that contrary to rigid polar rings of sugar-based surfactants, the polar heads of polyoxyethylene alkyl ethers are themselves flexible, their conformation may change with longer alkyl chains. Thus, the above analysis may not apply in their case.

When it comes to CMC, the alkyl chain length, as well-known for other surfactants [6], is the most relevant structural parameter. The dependence of the log CMC on the number of carbon atoms in the alkyl chain for two families, generally, obeys the expected empirical equation [21]:

(1) Log CMC=A−Bn

where n represents the carbon numbers (the length) in the alkyl chain; A and B are empirical constants, which are, respectively, related to the contributions of the hydrophilic head group and a single hydrophobic methylene group to the change in free energy for micellization. Plots of log CMC values as functions of the alkyl chain length are depicted in Figure 5 (bottom).

The results show that a linear relationship between the alkyl chain length and the log CMC exists for both ester families. For alkyl d-glucuronates derivatives (MeαGlc(C=O)OCn), the values of A and B calculated are 0.5 and 1.7, while for acyl d-glucosides derivatives (MeαGlcO(C=O)Cn), A is 0.5 and B is 1.5. The slightly higher B value in the case of alkyl d-glucuronates suggests a more rapid decrease in CMC when one carbon is added. It is important to note that the MeαGlc(C=O)OCn series shows even-numbered carbon atoms while the MeαGlcO(C=O)Cn series has odd-numbered carbon atoms in the noted Cn part. The amphiphilic molecules can be, in a simplistic way, depicted as the combination of a polar head group with a lipophilic tail. In the case of both families, the ester linkage is attributed to the polar moiety, even if it is not a part of the sugar: for acyl glycosides, this functional group is not located on a carbon atom of the sugar, whereas for the alkyl d-glucuronate, the saccharidic C6 is oxidized, leading to a difference in the structure (and particularly in the number of carbon atoms) of the polar head. We assume that this slight difference has a negligible effect on the hydrophilicity of the head group. On the other hand, the alkyl d-glucuronates series (MeαGlc(C=O)OCn) exhibits a slightly lower CMC and γcmc compared to the acyl d-glucosides series (MeαGlcO(C=O)Cn), which can be partially associated with the fact that the former always has one more carbon in its lipophilic moiety than the latter.

Some published CMC values are also worth comparing to the measurements from the present work. Otto et al. [20] obtained a CMC of 18 mM for 6-*O*-octanoyl-d-glucopyranose, noticeably higher than the CMC of MeαGlcO(C=O)C7 (9.8 mM). In addition, Blecker et al. [17] also reported a CMC of 10.68 mM for octyl d-glucuronate compared to the 6 mM value we observed for the methyl octyl α-d-glucuronate (MeαGlc(C=O)OC8). Both comparisons indicate that the nonpolar axial methyl group at the 1-position brings additional hydrophobicity to the polar head.

**Figure 6:**
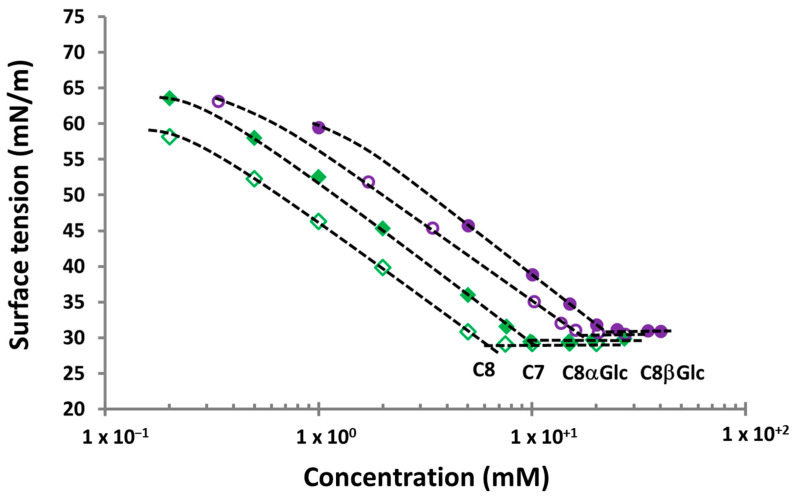
Surface tension as a function of the concentration for α-octyl glucoside (C8αGlc, ○) at 50 °C, β-octyl glucoside (C8βGlc, ●) at 25 °C, octyl α-methyl glucuronate (MeαGlc(C=O)OC8, ◇) at 25 °C and 6-octanoyl-α-methyl glucoside (MeαGlcO(C=O)C7, ◆) at 25 °C.

For the first time, Razafindralambo et al. [52] studied the effect of the ester orientation (Su-O-CO-R or Su-CO-O-R) on the surface properties of similar molecules: d-glucosyl octanoate and octyl d-glucuronate. Their analysis was that octyl d-glucuronate exhibits a stronger hydrophobic character because its carbonyl group was closer to the head group, making the head more polar and resulting in stronger intermolecular interactions between the heads. The CMC values at 25 °C for octyl d-glucuronate and d-glucosyl octanoate were reported to be 10.7 and 19.1 mM, respectively, which were almost twice as large as the CMC values for MeαGlc(C=O)OC8 and MeαGlcO(C=O)C7. It is worth noting that the Razafindralambo molecules are a mixture of both α and β anomers, with the β-d-glucose derivative being more hydrophilic. In addition, they obtained a larger Amin for octyl d-glucuronate, which was supported by a computational approach. Based on those results, the authors suggested that a shorter distance between the carbonyl group and the sugar head introduces a tilt between the polar head and the alkyl chain, resulting in looser packing at the water surface. For the synthesized surfactants of this work, however, our measurements do not evidence a significant influence of the ester orientation on Amin. It is possible that the anomeric methyl group dilutes the influence of the mechanism proposed by Razafindralambo et al. Nevertheless, their proposed mechanism itself remains questionable as the surface is a dynamic environment where molecules not only take different conformations but also different orientations. By comparing our results with other literature work, we note that the location of the ester group at the head group also influenced the CMC: sugar esters with the carbonyl group located at the 3-position appear more hydrophobic than those with the carbonyl group located at the 6-position. For example, Gouéth et al. [19] reported that 3-*O*-octanoyl-d-glucopyranose, which is a mixture of α and β anomers, had a CMC of 0.61 mM while Savellie et al. [21] reported a CMC of 1.6 mM. Both studies evidenced a lower CMC than that for 6-*O*-octanoyl-β-d-glucopyranose and our octyl derivative esters, as discussed above. Moreover, these molecules (MeαGlc-(C=O)OC8 and MeαGlcO(C=O)C7) show much lower CMC values compared to the commercial C8αGlc and C8βGlc, as shown in Figure 6. The results indicate that both the methyl group at the 1-position and the transformation of 6-OH into the ester group increase the hydrophobicity but do not influence γcmc significantly.

Our Amin measurements (see Table 2) indicate a more compact packing for the synthesized esters at the interface of the air–aqueous solution. This might be related to the stronger intermolecular interactions between their sugar heads due to the presence of the ester group.

**Figure 7:**
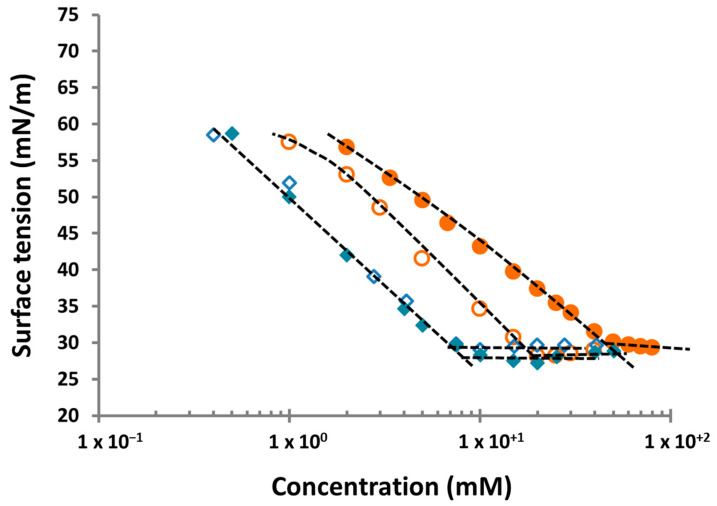
Surface tension as a function of concentration for β-octyl galactoside (C8βGal, ○) at 40 °C, α-octyl galactoside (C8αGal, ●) at 25 °C, octyl β-methyl galacturonate (MeβGal(C=O)OC8, ◇) at 40 °C and 6-octanoyl-β-methyl galactoside (MeβGalO(C=O)C7, ◆) at 25 °C.

In the case of MeβGal-based esters derivatives (Figure 7), the octyl d-galacturonate derivative (MeβGal(C=O)OC8) also showed a lower CMC than its homologous octanoyl d-galactoside derivative (MeβGalO(C=O)C7), which is consistent with MeαGlc-derived C7/C8 esters. Given that the actual alkyl chain of MeβGal(C=O)OC8 contains 8 carbons vs. 7 for MeβGalO(C=O)C7, and that the alkyl chain length is the major parameter relevant to CMC, the observed CMC difference is in line with expectations. It can also be noticed that the CMCs observed for the esters (6–9.8 mM) are lower than those of corresponding octyl d-galactosides (20–51.7 mM). Given the higher observed Amin for octyl d-galactosides, the shape of the esters’ polar heads may allow micellization more readily.

Overall, the main conclusions that arise by comparing the solubility and surface activity for all the synthesized C7/C8 esters are listed below:

− MeβGal-based esters show a much larger solubility limit than MeαGlc-based esters, suggesting that the solubility strongly depends on the head group configuration;
− Ester group orientation also influences the dissolution of MeβGal-based esters and MeαGlc-based esters;
− CMC is influenced by the ester orientation following the relationship: MeαGlc(C=O)OC8 < MeβGal(C=O)OC8 < MeβGalO(C=O)C7 < MeαGlcO(C=O)C7. The result suggests that the uronates derivatives exhibit lower CMC values than the acyl d-glycosides derivatives. The effect of ester group orientation on CMC was more evident for MeαGlc-derived esters than for MeβGal-derived esters;
− γcmc is very similar for all these esters, suggesting that the ester linkage orientation does not play an important role in the effectiveness of reducing surface tension.

## 3. Materials and Methods

### 3.1. General

Octyl α-d-glucopyranoside (>99%) and octyl β-d-glucopyranoside (>99%) were both purchased from Anatrace (Maumee, OH, USA). Octyl α-d-galactopyranoside and octyl β-d-galactopyranoside were purchased from Carbosynth (Compton, UK). They were all used without further purification. The deionized water with a resistivity of 18.2 Mcm was produced by a lab purification chain provided by Aquadem/Veolia Water STI (Saint-Maurice, France) and used to prepare all the solutions.

Alkyl (methyl d-glycopyranosid)uronates were synthesized using the two-step procedure developed in our lab, as previously described [29]. Briefly, the primary hydroxyl group of free methyl d-glycopyranosides was selectively and quantitatively oxidized by using 2,2,6,6-tetramethyl-1-piperidinyloxy free radical (TEMPO). Hydrophobic chains of different lengths were then introduced by acid-mediated esterification with fatty alcohols (hexyl to dodecyl alcohols) leading to the desired alkyl uronates with moderate to good yields (49–63%).

### 3.2. Synthesis of Methyl 6-O-acyl-d-glycopyranosides

#### 3.2.1. General

All chemicals were purchased from Fisher Scientific (Illkirch, France), and Merck Sigma Aldrich (St Quentin Fallavier, France), and used as received. Mass analyses were performed on a Waters spectrometer (SINAPT TG2SI, Manchester, UK) using electrospray ionization (Z-Spray). The NMR analysis was performed on a BRUKER NMR spectrometer (Bruker, Wissembourg, France) operating at 400 MHz for ^1^H and 100 MHz for ^13^C. Samples of silylated products were prepared in deuterated chloroform (CDCl_3_) while samples of free esters were dissolved in DMSO-*d*6.

#### 3.2.2. General Procedure for One-Pot Protection/Selective Deprotection

*N*,*O*-bis(trimethylsilyl)acetamide (28.7 g, 4.3 eq.) and tetrabutylammonium fluoride (235 mg, 0.03 eq.) were added to a solution of methyl α-d-glucopyranoside (9.30 g, 32.9 mmol) in dry pyridine (7.5 mL). After 1.5 h of stirring at room temperature, the reaction was quenched by adding isopropanol (6.25 mL) and methanol (450 mL). After cooling to 0 °C, K_2_CO_3_ (2.28 g, 0.5 eq.) was added, the mixture was stirred for 30 min at 0 °C, and then neutralized with 2.5 mL of acetic acid. After concentration, the crude was extracted with Et_2_O, washed with water, dried over Na_2_SO_4_, and concentrated to give methyl 2,3,4-tri-*O*-trimethylsilyl-α-d-glucopyranoside **1** as a white powder (10.94 g, yield 80%). The same procedure was applied to methyl β-d-galactopyranoside affording methyl 2,3,4-tri-*O*-trimethylsilyl-β-d-galactopyranoside **2** as a white powder (12.13 g, yield 90%).

#### 3.2.3. General Procedure for Esterification

To a solution of methyl 2,3,4-tri-*O*-trimethylsilyl-α-d-glycopyranoside **1** or **2** (7.00 g, 17.1 mmol) in anhydrous dichloromethane (80 mL), 4-dimethylaminopyridine (1.04 g, 0.5 eq.), desired fatty acid (37.5 mmol, 2.2 eq.), and 1-ethyl-3-(3-dimethylaminopropyl)carbodiimide (5.29 g, 2 eq.) were successively added and the mixture was stirred at room temperature for 6 h. After concentration, the crude was purified by normal phase column chromatography on silica gel (mobile phase: cyclohexane/EtOAc 9:1) to give corresponding silylated sugar esters **3**–**7**.

#### 3.2.4. General Procedure for Deprotection

To a solution of the as-obtained silylated ester glycoside, **3**–**7** (6 g) in CH_2_Cl_2_/MeOH 1:1 (100 mL), 3 g of Dowex-H^+^ resin was added. The suspension was stirred for 30 min at room temperature, then the resin was eliminated by filtration, and the filtrate was concentrated. The residue was purified by column chromatography on silica gel (mobile phase: EtOAc/MeOH 98:2) to give pure corresponding methyl 6-*O*-acyl d-glycosides **8**–**12**.

### 3.3. Compound Characterization Data

*Methyl 2,3,4-tri-O-trimethylsilyl-α-d-glucopyranoside* **1**. White powder. Yield 80% from methyl α-d-glucopyranoside following the general procedure. ^1^H and ^13^C NMR spectra in accordance with the literature [53].

*Methyl 2,3,4-tri-O-trimethylsilyl-β-d-galactopyranoside* **2**. White powder. Yield 90% from methyl β-d-galactopyranoside following the general procedure. ^1^H and ^13^C NMR spectra in accordance with the literature [54].

*Methyl 6-O-hexanoyl-2,3,4-tri-O-trimethylsilyl-α-d-glucopyranoside* **3**. Colorless syrup. Yield 72% from the methyl 2,3,4-tri-*O*-trimethylsilyl-α-d-glucopyranoside **1** following the general procedure. ^1^H NMR (400 MHz, CDCl_3_), δ 0.11–0.16 (m, 27H, (CH_3_)_3_-Si), 0.90 (t, *J*_CH3,CH2_ = 6.9 Hz 3H, (CH_2_)-CH_3_), 1.30–1.35 (m, 4H, CH_2_-(CH_2_)_2_-CH_3_), 1.60–1.68 (m, 2H, CO-CH_2_-CH_2_), 2.35 (o, *J* = 7.7 Hz, 2H, CO-CH_2_-CH_2_), 3.35 (s, 3H, O-CH_3_), 3.42–3.49 (m, 2H, H-2,H-5), 3.68–3.76 (m, 2H, H-3, H-4), 4.03 (dd, 1H, *J*_H-6,H-6′_ = 11.9 Hz, *J*_H-5,H-6′_ = 5.4 Hz H-6′), 4.40 (dd, 1H, *J*_H-6,H-6′_ = 11.9 Hz, *J*_H-5,H-6_ = 2.2 Hz, H-6), 4.70 (d, 1H, *J*_H-1,H-2_ = 3.7 Hz, H-1). ^13^C NMR (100 MHz, CDCl_3_), δ 0.5–1.5 (3s, (CH_3_)_3_-Si), 13.9 (CH_2_-CH_3_), 22.3–31.3 (2s, CH_2_-(CH_2_)_2_-CH_3_), 24.5 (CO-CH_2_-CH_2_-(CH_2_)_2_), 34.2 (CO-CH_2_-CH_2_), 54.8 (O-CH_3_), 63.3 (C-6), 69.4 (C-4), 72.5 (C-2), 73.7 (C-5), 75.1 (C-3), 99.8 (C-1), 173.6 (CO).

*Methyl 6-O-octanoyl-2,3,4-tri-O-trimethylsilyl-α-d-glucopyranoside* **4**. Colorless syrup. Yield 77% from the methyl 2,3,4-tri-*O*-trimethylsilyl-α-d-glucopyranoside **1** following the general procedure. ^1^H NMR (400 MHz, CDCl_3_) δ 0.10–0.15 (3 s, 27H, (CH_3_)_3_-Si), 0.85 (t, *J*_CH3,CH2_ = 6.9 Hz, 3H, CH_2_-CH_2_-CH_3_), 1.20–1.31 (m, 8H, CH_2_-(CH_2_)_4_-CH_3_), 1.58–1.63 (m, 2H, CO-CH_2_-CH_2_-CH_2_), 2.32 (m, 2H,CO-CH_2_-CH_2_), 3.32 (s, 3H, O-CH_3_), 3.42–3.48 (m, 2H, H-2,H-5), 3.67–3.75 (m, 2H, H-3, H-4), 4.02 (dd, 1H, *J*_H-6,H-6′_ = 11.9 Hz, *J*_H-5,H-6′_ = 5.4 Hz H-6′), 4.37 (d, 1H, *J*_H-6,H-6′_ = 11.8 Hz), 4.62 (d, 1H, *J*_H-1,H-2_ = 3.7 Hz, H-1). ^13^C NMR (100 MHz, CDCl_3_) δ 0–1.5 (3s, (CH_3_)_3_-Si), 14.0 (-CH_2_-CH_3_), 22.5–31.5 (4s, CH_2_-(CH_2_)_4_-CH_3_), 24.8 (CO-CH_2_-CH_2_-CH_2_), 34.2 (CO-CH_2_-CH_2_), 54.7 (O-CH_3_), 63.3 (C-6), 69.5 (C-4), 72.5 (C-2), 73.7 (C-5), 75.1 (C-3), 99.7 (C-1), 173.5 (CO).

*Methyl 6-O-decanoyl-2,3,4-tri-O-trimethylsilyl-α-d-glucopyranoside* **5**. Colorless syrup. Yield 88% from the methyl 2,3,4-tri-*O*-trimethylsilyl-α-d-glucopyranoside **1** following the general procedure. ^1^H NMR (400 MHz, CDCl_3_) δ 0.11–0.16 (3 s, 27H, (CH_3_)_3_-Si), 0.85 (t, *J*_CH3,CH2_ = 6.9 Hz, 3H, CH_2_-CH_3_), 1.19–1.35 (m, 12H, CH_2_-(CH_2_)_6_-CH_3_), 1.55–1.65 (m, 2H, CO-CH_2_-CH_2_), 2.37 (td, 2H, CO-CH_2_-CH_2_), 3.35 (s, 3H, O-CH_3_), 3.43–3.5 (m, 2H, H-2,H-5), 3.68–3.76 (m, 2H, H-3, H-4), 4.03 (dd, 1H, *J*_H-6,H-6′_ = 11,8 Hz, *J*_H-5,H-6′_ = 5.3 Hz, H-6′), 4.37 (dd, 1H, *J*_H-6,H-6′_ = 11.8 Hz, *J*_H-5,H-6_ = 2.3 Hz H-6), 4.60 (d, 1H, *J*_H-1,H-2_ = 3.7 Hz, H-1). ^13^C NMR (100 MHz, CDCl_3_) δ 0–1.5 (3s, (CH_3_)_3_-Si), 14.0 (-CH_2_-CH_3_), 22.5–32 (6s, CH_2_-(CH_2_)_6_-CH_3_), 24,7 (CO-CH_2_-CH_2_-CH_2_), 34.2 (CO-CH_2_-CH_2_), 55.0 (O-CH_3_), 63.2 (C-6), 69.4 (C-4), 72.5 (C-2), 73.8 (C-5), 75.0 (C-3), 99.8 (C-1), 173.5 (CO).

*Methyl 6-O-dodecanoyl-2,3,4-tri-O-trimethylsilyl-α-d-glucopyranoside* **6**. Colorless syrup. Yield 70% from the methyl 2,3,4-tri-*O*-trimethylsilyl-α-d-glucopyranoside **1** following the general procedure. ^1^H NMR (400 MHz, CDCl_3_) δ 0.14–0.19 (3s, 27H, (CH_3_)_3_-Si), 0.75 (t, *J*_CH3,CH2_ = 6.9 Hz, 3H, CH_2_-CH_2_-CH_3_), 1.20–1.30 (m, 16H, CH_2_-(CH_2_)_8_-CH_3_), 1.61–1.64 (m, 2H,CO-CH_2_-CH_2_-CH_2_), 2.35 (td, 2H,CO-CH_2_), 3.35 (s, 3H, O-CH_3_), 3.43–3.49 (m, 2H, H-2, H-5), 3.68–3.78 (m, 2H, H-3, H-4),4.05 (dd, 1H, *J*_H-6,H-6′_ = 11.8 Hz, *J*_H-5,H-6′_ = 5.4 Hz, H-6′), 4.39 (dd, 1H, *J*_H-6,H-6′_ = 11.8 Hz, *J*_H-5,H-6_ = 2.2 Hz, H-6), 4.62 (d, 1H, *J*_H-1,H-2_ = 3.6 Hz, H-1). ^13^C NMR (100 MHz, CDCl_3_) δ 0–1.5 (3s, (CH_3_)_3_-Si), 14.0 (-CH_2_-CH_3_), 22.5–21.5 (8s, CH_2_-(CH_2_)_8_-CH_3_), 24.5 (CO-CH_2_-CH_2_), 34.3 (CO-CH_2_), 55.0 (O-CH_3_), 63.2 (C-6), 69.5 (C-4), 72.6 (C-2), 73.8 (C-5), 75.0 (C-3), 99.8 (C-1), 173.4 (CO).

*Methyl 6-O-octanoyl-2,3,4-tri-O-trimethylsilyl-β-d-galactopyranoside* **7**. Colorless syrup. Yield 83% from the methyl 2,3,4-tri-*O*-trimethylsilyl-β-d-galactopyranoside **2** following the general procedure. ^1^H NMR (400 MHz, CDCl_3_) δ 0.12 (3s, 27H (CH_3_)_3_-Si), 0.85 (t, *J*_CH3,CH2_ = 6.9 Hz, 3H, CH_2_-CH_2_-CH_3_), 1.20–1.31 (m, 8H, CH_2_-(CH_2_)_4_-CH_3_), 1.61 (p, 2H, *J* = 7.4 Hz CO-CH_2_-CH_2_-CH_2_), 2.30 (t, 2H, *J* = 7.5 Hz, CO-CH_2_-CH_2_), 3.40 (dd, 1H, *J*_H-2,H-3_ = 9.3 Hz, *J*_H-3,H-4_ = 2.7 Hz, H-3), 3.47 (s, 3H, O-CH_3_), 3.64 (m, 2H, H-2,H-5), 3.74 (d, 1H, *J*_H-3,H-4_ = 2.3 Hz, H-4), 4.06 (d, 1H, *J*_H-1,H-2_ = 7.5 Hz, H-1), 4.13 (dd, 1H, *J*_H-6,H-6′_ = 11.0 Hz, *J*_H-5,H-6_ = 6.5 Hz, H-6), 4.23 (dd, 1H, *J*_H-6,H-6′_ = 11.0 Hz, *J*_H-5,H-6′_ = 6.6 Hz, H-6′), ^13^C NMR (100 MHz, CDCl_3_) δ 0.6, 0.7, 0.8 (3s, (CH_3_)_3_-Si), 14.2 (-CH_2_-CH_3_), 22.7, 29.0, 29.2, 31.7 (4s, CH_2_-(CH_2_)_4_-CH_3_), 25.1 (CO-CH_2_-CH_2_-CH_2_), 34.4 (CO-CH_2_-CH_2_), 57.2 (O-CH_3_), 63.0 (C-6), 71.9 (C-4), 72.0 (C-2), 72.2 (C-5), 75.1 (C-3), 105.0 (C-1), 173.6 (CO).

*Methyl 6-O-hexanoyl-α-d-glucopyranoside* **8**. Colorless syrup. Yield 77% from the methyl 6-*O*-hexanoyl-2,3,4-tri-*O*-trimethylsilyl-α-d-glucopyranoside **3** following the general procedure for deprotection. ^1^H NMR (400 MHz, DMSO-*d*6) δ 0.84 (t, 3H, *J*_CH3,CH2_ = 6.9 Hz, CH_2_-CH_3_), 1.21–1.29 (m, 4H, -(CH_2_)_2_-CH_3_), 1.44–1.57 (m, 2H, CO-CH_2_-CH_2_), 2.28 (t, 2H, *J*_CH2,CH2_ = 7.3 Hz, CO-CH_2_), 3.02–3.09 (m, 1H, H-4) 3.16–3.23 (m,1H, H-2) 3.25 (s, 3H, O-CH_3_), 3.35–3.41 (m, 1H, H-3), 3.48–3.53 (m, 1H, H-5) 4.01 (dd, 1H, *J*_H-6,H-6′_ = 11.6 Hz, *J*_H-5,H-6′_ = 6.5 Hz, H-6′), 4.29 (dd, 1H, *J*_H-6,H-6′_ = 11.6 Hz, *J*_H-5,H-6_ = 2.1 Hz, H-6), 4.52 (d, 1H, *J*_H-1,H-2_ = 3.7 Hz, H-1) 4.82 (d, 1H, *J*_OH,H-2′_ = 6.4 Hz, OH-2), 4.90 (d, 1H, *J*_OH,H-3’_ = 5.0 Hz, OH-3), 5.15 (d, 1H, *J*_OH, H-4’_ = 5.8 Hz, OH-4). ^13^C (100 MHz, DMSO-*d*6) δ 13.9 (-CH_2_-CH_3_), 22.9, 24.3, 30.7 (3 s, -(CH_2_)_3_-CH_3_), 33.6 (CO-CH_2_-), 54.5 (O-CH_3_), 63.6 (C-6), 69.7 (C-5), 70.5 (C-4), 71.9 (C-2), 73.3 (C-3), 99.8 (C-1), 173.0 (CO). HRMS (ESI) *m*/*z* for C_13_H_24_O_7_Na^+^ [M + Na]^+^ calcd 315.1420, found 315.1424.

*Methyl 6-O-octanoyl-α-d-glucopyranoside* **9**. White powder. Yield 77% from the methyl 6-*O*-octanoyl-2,3,4-tri-*O*-trimethylsilyl-α-d-glucopyranoside **4** following the general procedure for deprotection. ^1^H NMR (400 MHz, DMSO-*d*6) δ 0.84 (t, 3H, *J*_CH3,CH2_ = 7.3 Hz, CH_2_-CH_3_), 1.17–1.30 (m, 8H, -(CH_2_)_4_-CH_3_), 1.47–1.55 (m, 2H, CO-CH_2_-CH_2_), 2.28 (t, 2H, *J*_CH2,CH2_ = 7.3 Hz, CO-CH_2_), 3.02–3.09 (m, 1H, H-4) 3.16–3.23 (m, 1H, H-2) 3.25 (s, 3H, O-CH_3_), 3.35–3.41 (m, 1H, H-3), 3.50–3.56 (m, 1H, H-5) 4.01 (dd, 1H, *J*_H-6,H-6′_ = 11.7 Hz, *J*_H-5,H-6′_ = 6.7 Hz, H-6′), 4.28 (dd, 1H, *J*_H-6,H-6′_ = 11.7 Hz, *J*_H-5,H-6_ = 1.9 Hz, H-6), 4.52 (d, 1H, *J*_H-1,H-2_ = 3.6 Hz, H-1) 4.82 (d, 1H, *J*_OH,H-2′_ = 6.4 Hz, OH-2), 4.90 (d, 1H, *J*_OH,H-3’_ = 4.9 Hz, OH-3), 5.15 (d, 1H, *J*_OH,H-4’_ = 5.8 Hz, OH-4). ^13^C (100 MHz, DMSO-*d*6) δ 14.1 (-CH_2_-CH_3_), 22.2, 24.6, 28.5, 28.5, 31.2 (5 s, (CH_2_)_5_-CH_3_), 33.6 (CO-CH_2_-), 54.4 (O-CH_3_), 63.6 (C-6), 69.7 (C-5), 70.5 (C-4), 71.9 (C-2), 73.3 (C-3), 99.8 (C-1), 173.0 (CO). HRMS (ESI) *m*/*z* for C_15_H_28_O_7_Na^+^ [M + Na]^+^ calcd 343.1733, found 343.1726.

*Methyl 6-O-decanoyl-α-d-glucopyranoside* **10**. White powder. Yield 81% from the methyl 6-*O*-decanoyl-2,3,4-tri-*O*-trimethylsilyl-α-d-glucopyranoside **5** following the general procedure for deprotection. ^1^H NMR (400 MHz, CD_3_OD) δ 0.89 (t, 3H, *J*_CH3,CH2_ = 7.3 Hz, CH_2_-CH_3_), 1.24–1.39 (m, 12H, -(CH_2_)_6_-CH_3_), 1.62 (p, 2H, *J*_CH2,CH2_ = 7.2 Hz, CO-CH_2_-CH_2_), 2.35 (t, 2H, *J*_CH2,CH2_ = 7.4 Hz, CO-CH_2_-CH_2_), 3.27 (dd, 1H, *J*_H-3,H-4_ = 9.0 Hz, *J*_H-4,H-5_ = 9.9 Hz, H-4) 3.37–3.42 (m, 4H, H-2, O-CH_3_), 3.61 (t, 1H, *J*_H-2,H-3_ = *J*_H-3,H-4_ = 9.3 Hz, H-3), 3.70 (ddd, 1H, *J*_H-4,H-5_ = 9.9 Hz, *J*_H-5,H-6_ = 2.0 Hz, *J*_H-5,H-6′_ = 6.0 Hz, H-5), 4.19 (dd, 1H, *J*_H-6,H-6′_ = 11.8 Hz, *J*_H-5,H-6′_ = 6.0 Hz, H-6′), 4.38 (dd, 1H, *J*_H-6,H-6′_ = 11.8 Hz, *J*_H-5,H-6_ = 2.1 Hz, H-6), 4.65 (d, 1H, *J*_H-1,H-2_ = 3.7 Hz, H-1). ^13^C (100 MHz, CD_3_OD) δ 14.5 (-CH_2_-CH_3_), 23.7, 26.1, 30.2, 30.4, 30.4, 30.6, 33.0 (7 s, -(CH_2_)_7_-CH_3_), 35.0 (CO-CH_2_-), 55.6 (O-CH_3_), 64.7 (C-6), 71.0 (C-5), 71.9 (C-4), 73.4 (C-2), 75.0 (C-3), 101.3 (C-1), 175.4 (CO). HRMS (ESI) *m*/*z* for C_17_H_32_O_7_Na^+^ [M + Na]^+^ calcd 371.2046, found 371.2046.

*Methyl 6-O-dodecanoyl-α-d-glucopyranoside* **11**. White powder. Yield 71% from the methyl 6-*O*-dodecanoyl-2,3,4-tri-*O*-trimethylsilyl-α-d-glucopyranoside **6** following the general procedure for deprotection. ^1^H NMR (400 MHz, CD_3_OD) δ 0.89 (t, 3H, *J*_CH3,CH2_ = 7.3 Hz, CH_2_-CH_3_), 1.24–1.40 (m, 16H, -(CH_2_)_8_-CH_3_), 1.62 (p, 2H, *J*_CH2,CH2_ = 7.2 Hz, -CO-CH_2_-CH_2_), 2.35 (t, 2H, *J*_CH2,CH2_ = 7.4 Hz, CO-CH_2_), 3.27 (dd, 1H, *J*_H-3,H-4_ = 9.0 Hz, *J*_H-4,H-5_ = 10.0 Hz, H-4) 3.37–3.42 (m, 4H, H-2, O-CH_3_), 3.61 (t, 1H, *J*_H-2,H-3_ = *J*_H-3,H-4_ = 9.3 Hz, H-3), 3.70 (ddd, 1H, *J*_H-4,H-5_ = 9.9 Hz, *J*_H-5,H-6_ = 2.0 Hz, *J*_H-5,H-6′_ = 6.0 Hz, H-5), 4.19 (dd, 1H, *J*_H-6,H-6′_ = 11.8 Hz, *J*_H-5,H-6′_ = 6.0 Hz, H-6′), 4.38 (dd, 1H, *J*_H-6,H-6′_ = 11.8 Hz, *J*_H-5,H-6_ = 2.1 Hz, H-6), 4.65 (d, 1H, *J*_H-1,H-2_ = 3.7 Hz, H-1). ^13^C (100 MHz, CD_3_OD) δ 14.5 (-CH_2_-CH_3_), 23.8, 26.1, 30.2, 30.4, 30.5, 30.6, 30.8, 30.8, 33.1 (9 s, -(CH_2_)_9_-CH_3_), 35.0 (CO-CH_2_-), 55.6 (O-CH_3_), 64.7 (C-6), 71.1 (C-5), 71.9 (C-4), 73.5 (C-2), 75.0 (C-3), 101.3 (C-1), 175.4 (CO). HRMS (ESI) *m*/*z* for C_19_H_36_O_7_Na^+^ [M + Na]^+^ calcd 399.2359, found 399.2361.

*Methyl 6-O-octanoyl-β-d-galactopyranoside* **12**. White powder. Yield 83% from the methyl 6-*O*-octanoyl-2,3,4-tri-*O*-trimethylsilyl-β-d-galactopyranoside **7** following the general procedure for deprotection. ^1^H NMR (400 MHz, CD_3_OD) δ 0.89 (t, 3H, *J*_CH3,CH2_ = 7.3 Hz, CH_2_-CH_3_), 1.26–1.37 (m, 8H, CH_2_-(CH_2_)_4_-CH_3_), 1.57–1.65 (m, 2H, CH_2_-CH_2_-(CH_2_)_4_), 2.34 (t, 2H, *J* = 7.4 Hz, CO-CH_2_-CH_2_), 3.44–3.51 (m, 2H, H-2, H-3) 3.50 (s, 3H, O-CH_3_), 3.71 (ddd, 1H, J_H-5,H-6_ = 7.3 Hz, J_H-5,H-6′_ = 5.0 Hz, J_H-4,H-5_ = 1.0 Hz, H-5), 3.79 (d, 1H, *J*_H-3,H-4_ = 2.0 Hz, H-4), 4.12 (d, 1H, *J*_H-1,H-2_ = 7.5 Hz, H-1), 4.21 (dd, 1H, *J*_H-6,H-6′_ = 11.4 Hz, *J*_H-5,H-6_ = 5.0 Hz, H-6), 4.30 (dd, 1H, *J*_H-6,H-6′_ = 11.3 Hz, *J*_H-5,H-6′_ = 7.4 Hz, H-6′), ^13^C NMR (100 MHz, CD_3_OD) δ 14.4 (-CH_2_-CH_3_), 23.7, 26.1, 30.1, 30.2, 32.8 (5 s, -(CH_2_)_5_-CH_3_), 35.0 (CO-CH_2_-), 57.2 (O-CH_3_), 64.6 (C-6), 70.3 (C-4), 72.3 (C-2), 73.9 (C-5), 74.7 (C-3), 105.9 (C-1), 175.3 (CO). HRMS (ESI) *m*/*z* for C_15_H_28_O_7_Na^+^ [M + Na]^+^ calcd 343.1733, found 343.1743.

### 3.4. Physicochemical Characterizations

#### 3.4.1. Solubility

Water solubility reflects the ability of a particular molecule to dissolve and its interaction with water. To characterize the water solubility of most amphiphile molecules, especially the ionic surfactants, the Krafft point (Tk) is often used, referring to the critical temperature above which micelles form or the melting point of the hydrated solid surfactant. Two general approaches were used to measure the Tks of the molecules investigated herein: (1) a visual examination of the dissolution phenomenon for binary molecule/water systems, and (2) a quantitative measurement by using differential scanning calorimetry (DSC) analysis. The visual observation procedure is detailed below. We start with adding the molecule into 5–50 mL water, in a concentration range between 10^−2^ mM and 10^3^ mM (corresponding to a range of a surfactant mass concentration well below 20%), keeping the suspension under stirring for a certain time at room temperature (RT). Consequently, molecules can be classified into two major types: those soluble in water at RT and others insoluble at RT, with the solubility limit being called S (expressed in mM). The aspect of the sample, either transparent or turbid, is determined by direct visual examination. Tk determination with DSC is particularly applicable in the case of molecules insoluble at RT, for which a concentrated binary molecule/water mixture with a mass fraction of 20% of each molecule (C >> CMC in most cases) was prepared at 65 °C under stirring for 30 min to obtain a homogeneous system. These pre-homogenized aqueous systems were cooled and conserved in a refrigerator at 4 °C for a suitable period (>7 days), during which equilibrated hydrated crystals formed and precipitated. Then, for each molecule, about a 200–400 mg crystal sample was taken and put into the calorimeter cell. The first equilibrium phase was programmed at 4 °C for 1 h, followed by a slow heating stage from 4 °C to 65 °C at a rate of 0.5 °C/min. Tk was determined based on the location of the endothermic peak on the DSC curve, if any. All agitations were performed using a standard magnetic hotplate stirrer, MR 3001K (Heidolph Instruments, Schwabach, Germany). DSC curves were obtained from a commercial µDSC7 evo calorimeter (SETARAM Instrumentation, Caluire-et-Cuire, France). Tks are reported as the averages of at least two measurements as well as their standard deviations.

#### 3.4.2. Surface Activity

The surface activity reflects the amphiphiles’ adsorption behavior on the air–aqueous solution interface. The surface tension (*γ*) was obtained using the Wilhelmy plate method with a K100 Processor Tensiometer (KRUSS, Hamburg, Germany). Both the critical micellar concentration (CMC) and equilibrium surface tension at the CMC (γcmc) were derived from the typical plot of surface tension (*γ*) against the logarithm of concentration (Log C). The surface excess (Γmax) and minimal area per molecule at the air–aqueous solution interface (Amin) at different temperatures were calculated from the Gibbs adsorption equation [3,46]:

(2) $$\Gamma_{max}=-\frac{1}{2.303RT}(\frac{d\gamma }{dlogc})$$

and

(3) $$A_{min}=\frac{10^{20}}{N_{A}\Gamma_{max}}$$

where *R* is the ideal gas constant, 8.31 J∙mol^−1^∙K^−1^; *T* is the measuring temperature, K; *γ* is the surface tension, N/m; *c* is the concentration, mol.L^−1^; A_min_ is the minimal area per molecule, Å^2^, and N_A_ is Avogadro’s number, 6.022·10^23^ mol^−1^.

For each molecule, several solutions of a wide range of concentrations were prepared. Before measuring, each solution was finely homogenized with the help of a magnetic stirring bar. The stirring time was fixed at 30 min in all cases. For molecules with a Tk below RT, the preparation was performed at environmental conditions, whereas for molecules exhibiting a Tk higher than 25 °C, the aqueous mixtures were firstly heated and agitated at elevated temperatures (25–50 °C), depending on the corresponding Tk. Then, measurements were performed once for each concentration, at the solution preparation’s temperature, with an uncertainty of ±1 °C.

## 4. Conclusions

Two series of methyl d-glycopyranoside-based esters, with different orientations of the ester group, were compared in terms of solubility and surface adsorption properties. This study, combined with a comparison of four well-studied commercial octyl glycosides (C8αGlc, C8βGlc, C8αGal, and C8βGal), allows for a careful exploration of how structural features affect their physicochemical properties.

Commercial octyl d-glycosides presented high solubility limits although their Krafft points were strongly influenced by the head group configuration. Only C8αGlc and C8βGal were found to have Tks above room temperature. Specific structural features causing differences in solubility were proposed for these molecules. For sugar esters families, the ester group orientation had an obvious impact on the dissolution behavior of the compounds.

With respect to the surface tension measurements, we observed, for both series of ester surfactants, a clear linear decrease in the log CMC with the alkyl chain length, in line with well-established surfactant science research. In addition, we could also evidence a slight decrease in γcmc and Amin with alkyl chain lengths, consistent with literature-based expectations for sugar-based surfactants. By comparing the commercial octyl d-glycosides and all the synthesized C7/C8 esters, we found that the latter exhibits a noticeably lower CMC. At a constant alkyl chain length, the various polar heads studied in this work did not seem to impact γcmc significantly.

Overall, this work offers a refined understanding of the various factors involved in the physicochemical properties of sugar-based amphiphiles. While being able to predict structure-amphiphilic property trends should allow the design of greener surfactants in a smarter way, it is obvious that predicting the solubility properties of new amphiphilic molecules is tricky and challenging, as sugar-based surfactant solubility arises from a delicate balance between three key factors—their crystal stability, hydrophilicity, and self-assembling tendency. Further research on the structure–property relationships of sugar-based surfactants are still needed to contribute to community knowledge and to provide significant hope regarding the replacement of petroleum-based substances.

## Figures and Tables

**Table 1:** Physical parameters related to the solubility properties (Krafft temperatures, solubility limits) for all studied molecules.

Family	Molecule	Tk (°C)	Solubility Limit (S/mM)	State
Alkyl glycosides	C8αGlc	39 ± 1	insoluble at RT; >100 at T > Tk	Powder
	C8βGlc	<4	>100 at RT	Powder
	C8αGal	<4	>100 at RT	Highly viscous liquid
	C8βGal	29.0 ± 0.5	10 < S < 20 at RT; >100 at T > Tk	Powder
Alkyl uronates	MeαGlc(C=O)OC6	<4	≤50 at RT	Wax
	MeαGlc(C=O)OC8	<4	15~20 at RT	Wax
	MeαGlc(C=O)OC10	<4	0.5~0.75 at RT	Powder
	MeαGlc(C=O)OC12	22.1 ± 0.2	0.02~0.05 at RT	Powder
	MeβGal(C=O)OC8	31.6 ± 0.9	insoluble at RT; >100 at T > Tk	Powder
Acyl glycosides	MeαGlcO(C=O)C5	<4	<40 at RT	Oil
	MeαGlcO(C=O)C7	<4	≤10 at RT	Powder
	MeαGlcO(C=O)C9	26.5 ± 0.2	insoluble at RT; ≤1 at T > Tk	Powder
	MeαGlcO(C=O)C11	42.9 ± 0.1	insoluble at RT; ≤0.1 at T > Tk	Powder
	MeβGalO(C=O)C7	<4	>100 at RT	Powder

**Table 2:** Surface tension data at T > Tk for different molecules.

Family	Molecule	CMC (mM)	γcmc (mN/m)	Amin (Å^2^/molecule)	ΔG_mic_ (kcal/mol)	ΔG_ads_ (kcal/mol)	C20 (mM)	pC20	T (°C)
Alkyl glucosides	C8αGlc	15.5 ± 0.5	30.7 ± 0.3	43 ± 1.8	−4.7	−7.0	2.8	2.6	50
	C8βGlc	21.2 ± 0.8	31.0 ± 0.15	38.5 ± 1.9	−4.1	−6.4	2.7	2.6	25
	C8αGal	51.7 ± 1.5	29.5 ± 0.2	45.8 ± 0.9	−3.6	−6.4	3.8	2.4	25
	C8βGal	20 ± 1	28.5 ± 0.5	46.3 ± 1.7	−4.4	−7.1	3.2	2.5	40
Alkyl uronates	MeαGlc(C=O)OC6	55 ± 5	30.8 ± 0.8	39.2 ± 2.9	−3.6	−5.9	5.9	2.2	25
	MeαGlc(C=O)OC8	6 ± 0.5	29.2 ± 0.35	41 ± 1	−4.8	−7.3	0.5	3.3	25
	MeαGlc(C=O)OC10	0.65 ± 0.5	28.1 ± 0.1	42.4 ± 2	−5.9	−8.6	0.05	4.3	25
	MeαGlc(C=O)OC12	0.056 ± 0.004	28.3 ± 0.3	45.6 ± 5	−7.2	−10.1	0.004	5.4	25
	MeβGal(C=O)OC8	6.9 ± 0.4	29.4 ± 0.36	42.9 ± 3.9	−5.0	−7.4	1.2	2.9	40
Acyl glycosides	MeαGlcO(C=O)C5	105 ± 5	31 ± 0.1	40.1 ± 0.3	−3.3	−5.6	11.2	2.0	25
	MeαGlcO(C=O)C7	9.8 ± 0.76	29.5 ± 0.5	39 ± 2	−4.5	−6.9	1.0	3.0	25
	MeαGlcO(C=O)C9	0.92 ± 0.08	27.9 ± 0.05	41.8 ± 0.9	−5.8	−8.4	0.08	4.1	30
	MeαGlcO(C=O)C11	0.08 ± 0.005	26.7 ± 0.25	45.8 ± 2.8	−7.7	−10.4	0.007	5.1	50
	MeβGalO(C=O)C7	8.5 ± 0.5	28.3 ± 0.35	42.7 ± 2.4	−4.6	−7.3	0.7	3.1	25

## Data Availability

The original contributions presented in the study are included in the article/Supplementary Materials, further inquiries can be directed to the corresponding author/s.

## Supplementary Materials

The following supporting information can be downloaded at https://www.mdpi.com/article/10.3390/molecules29102338/s1, ^1^H and ^13^C NMR spectra of final compounds (**8**–**12**).
