# Supplementary material for: Sugar-Based Surfactants: Effects of Structural Features on the Physicochemical Properties of Sugar Esters and Their Comparison to Commercial Octyl Glycosides
Source: Molecules. 2024 May 16;29(10):2338. doi: 10.3390/molecules29102338 (PMC11123692; doi:10.3390/molecules29102338)
Supplement: Supplementary file 1 [file molecules-29-02338-s001.zip › molecules-2953403-supplementary.pdf]

Supplementary Materials: Sugar-Based Surfactants: Effects of Structural Features on the Physicochemical Properties of Sugar Esters and their Comparison to Commercial Octyl Glycosides

Huiling Lu [1], Gwladys Pourceau [2,*], Benoit Briou [2], Anne Wadouachi [2], Théophile Gaudin, [1] Isabelle Pezron [1] and Audrey Drelich [1,*]

[1] Université de Technologie de Compiègne, ESCOM, TIMR (Transformations Intégrées de la Matière Renouvelable), Centre de Recherche Royallieu—CS 60 319, 60203 Compiègne Cedex, France; lhl@ujs.edu.cn (H.L.); isabelle.pezron@utc.fr (I.P.)

[2] Laboratoire de Glycochimie, et des Agroressources d'Amiens (LG2A), UR 7378—Institut de Chimie de Picardie, Université de Picardie Jules Verne, 33 rue Saint Leu, 80039 Amiens Cedex, France; b.briou@orpiainnovation.com (B.B.); anne.wadouachi@u-picardie.fr (A.W.)

* Correspondence: gwladys.pourceau@u-picardie.fr (G.P.); audrey.drelich@utc.fr (A.D.)

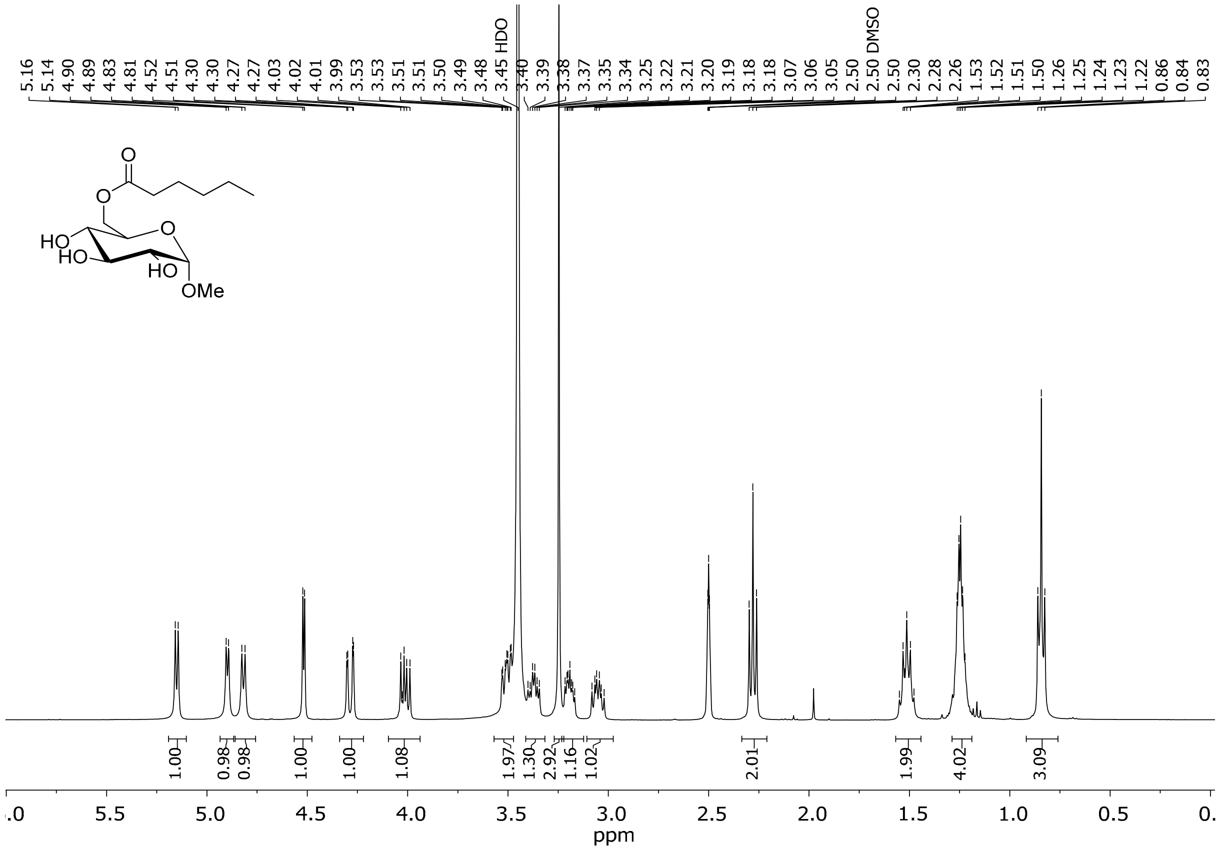

Figure S1. ^{1}H-NMR spectrum of methyl 6-*O*-hexanoyl-α-D-glucopyranoside **8**.

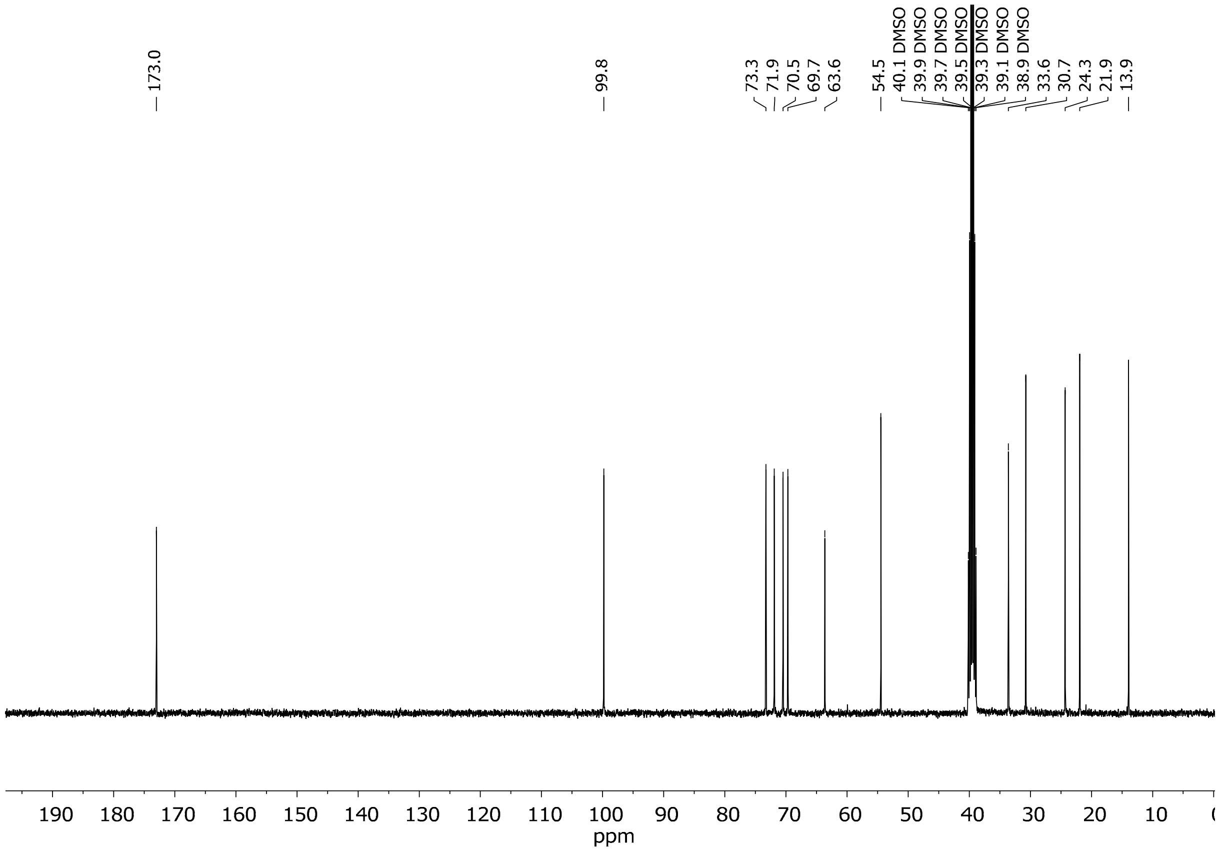

Figure S2. ^{13}C-NMR spectrum of methyl 6-*O*-hexanoyl-α-D-glucopyranoside **8**.

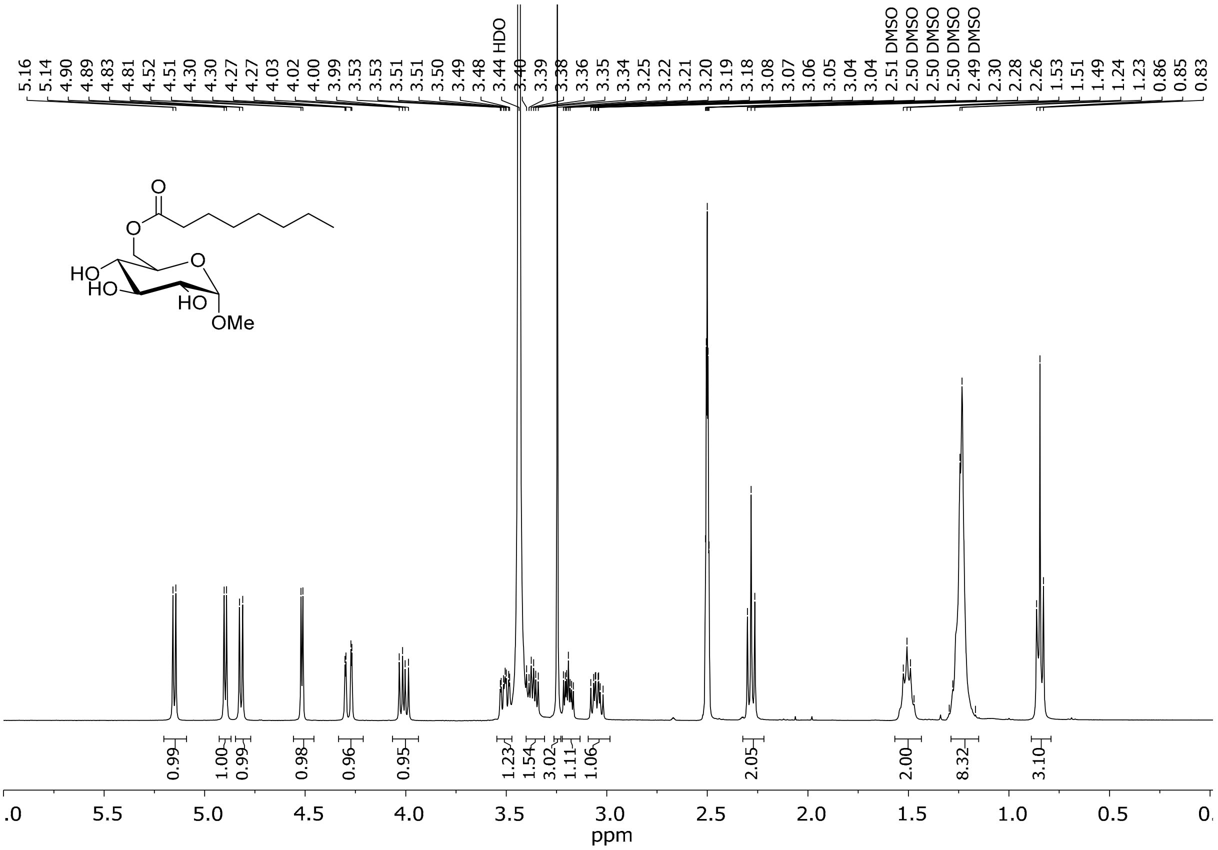

Figure S3. ^{1}H-NMR spectrum of methyl 6-*O*-octanoyl-α-D-glucopyranoside **9**.

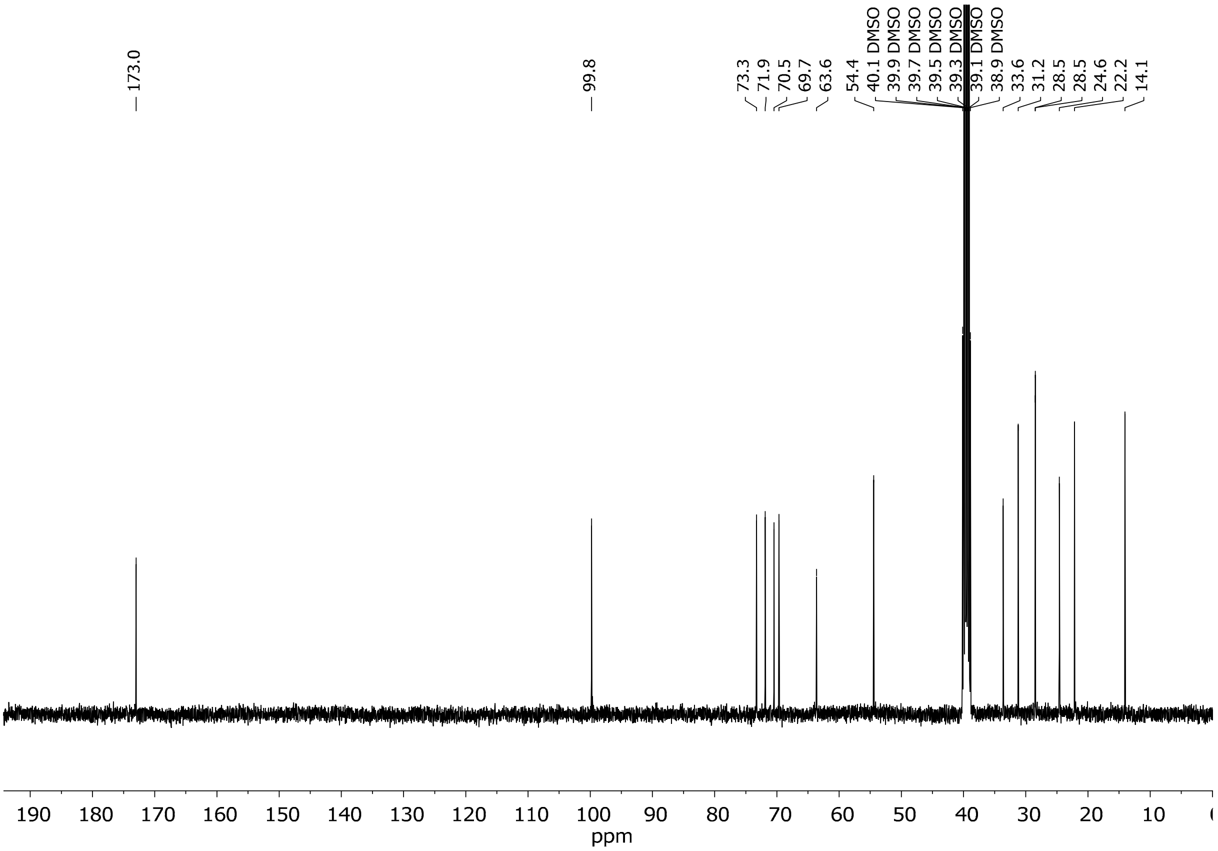

Figure S4. ^{13}C-NMR spectrum of methyl 6-*O*-octanoyl-α-D-glucopyranoside **9**.

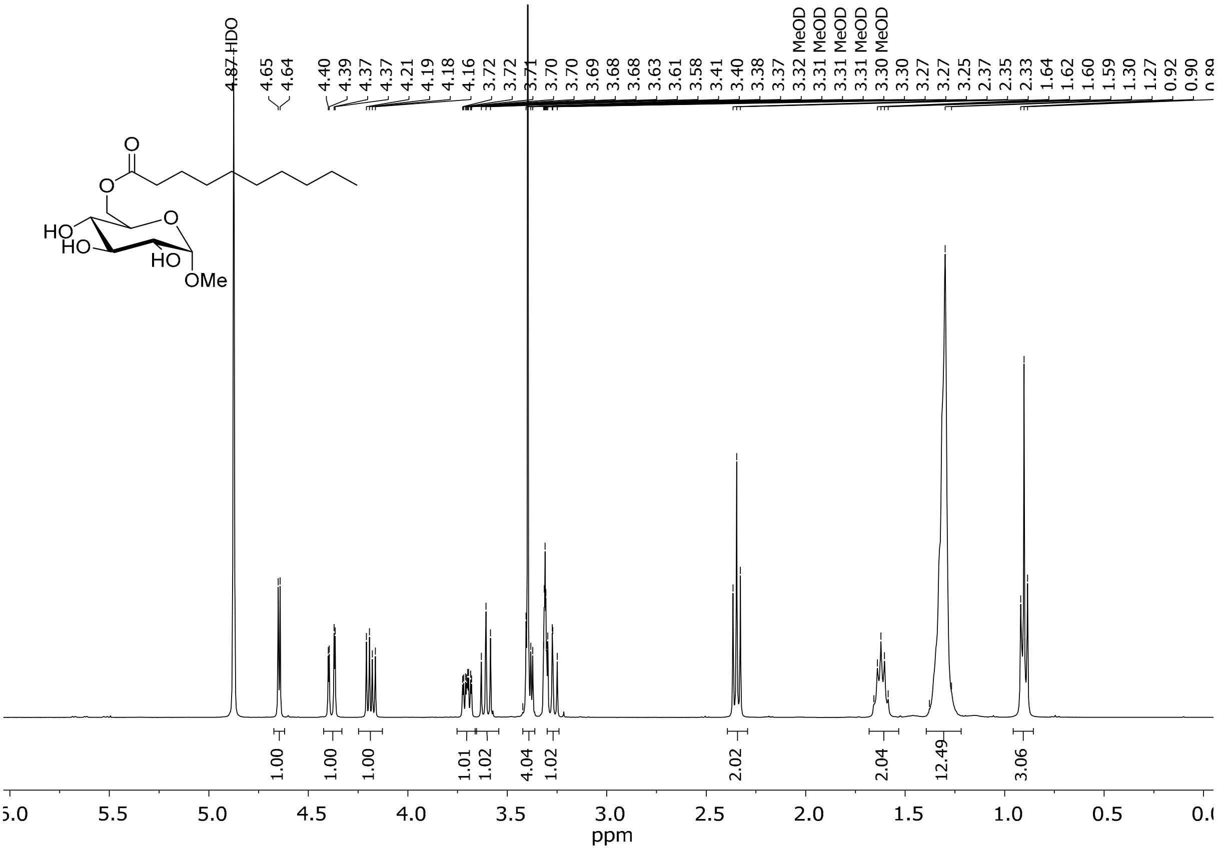

Figure S5. ^{1}H-NMR spectrum of methyl 6-*O*-decanoyl-α-D-glucopyranoside **10**.

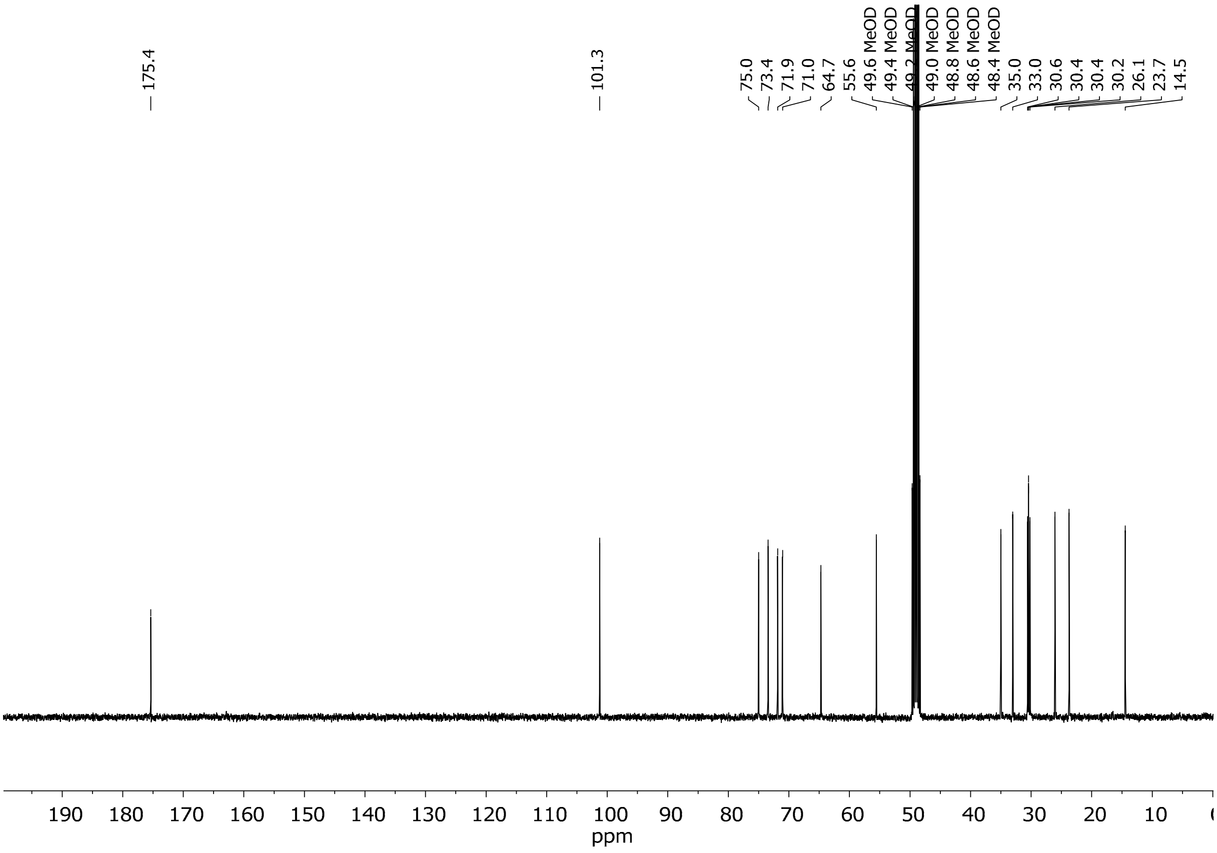

Figure S6. ^{13}C-NMR spectrum of methyl 6-*O*-decanoyl-α-D-glucopyranoside **10**.

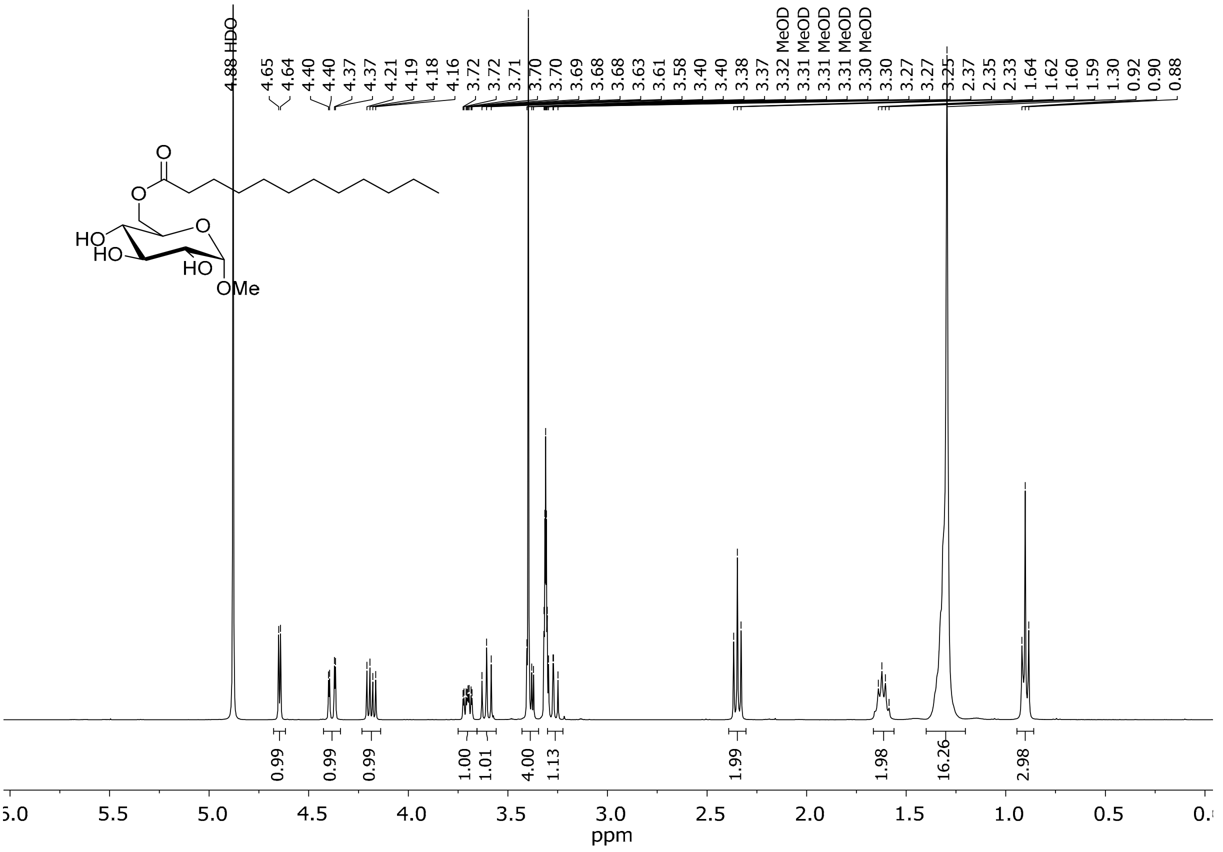

Figure S7. ^{1}H-NMR spectrum of methyl 6-*O*-dodecanoyl-α-D-glucopyranoside **11**.

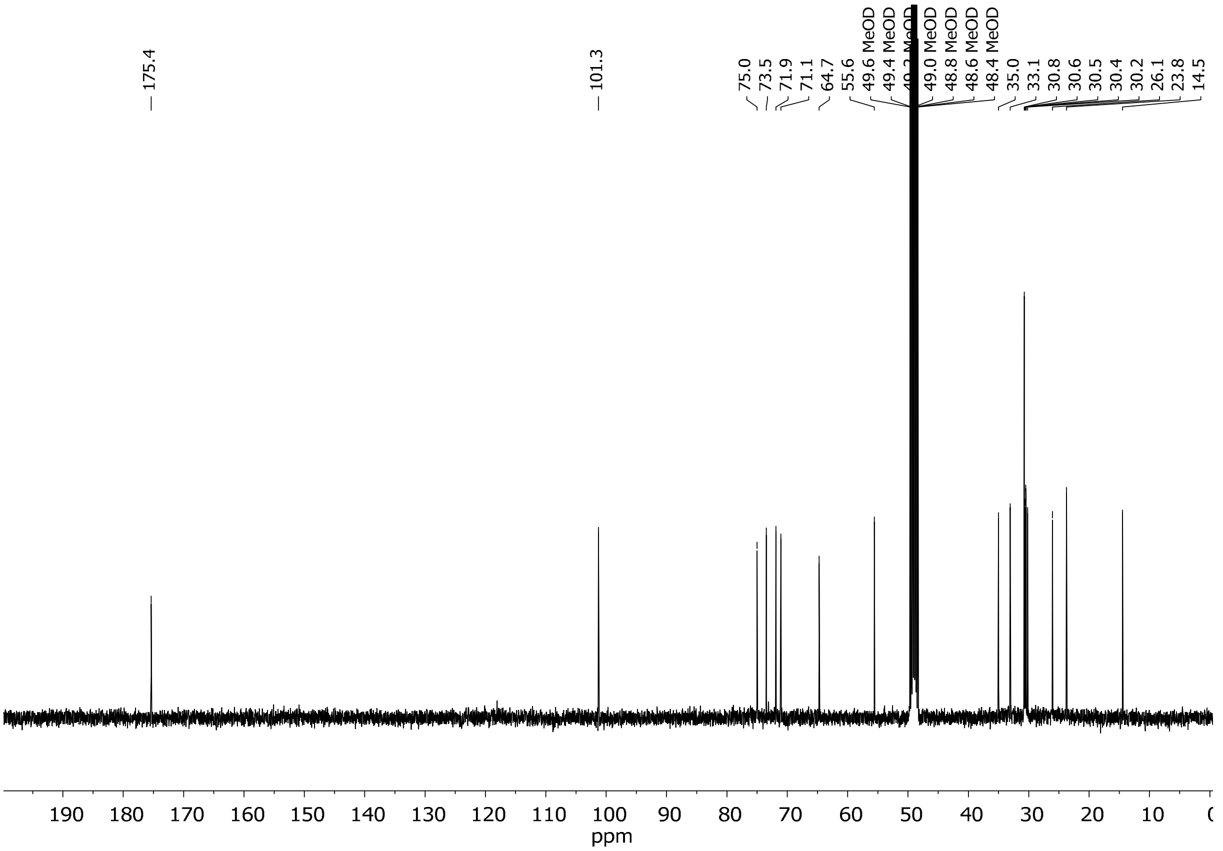

Figure S8. ^{13}C-NMR spectrum of methyl 6-*O*-dodecanoyl-α-D-glucopyranoside **11**.

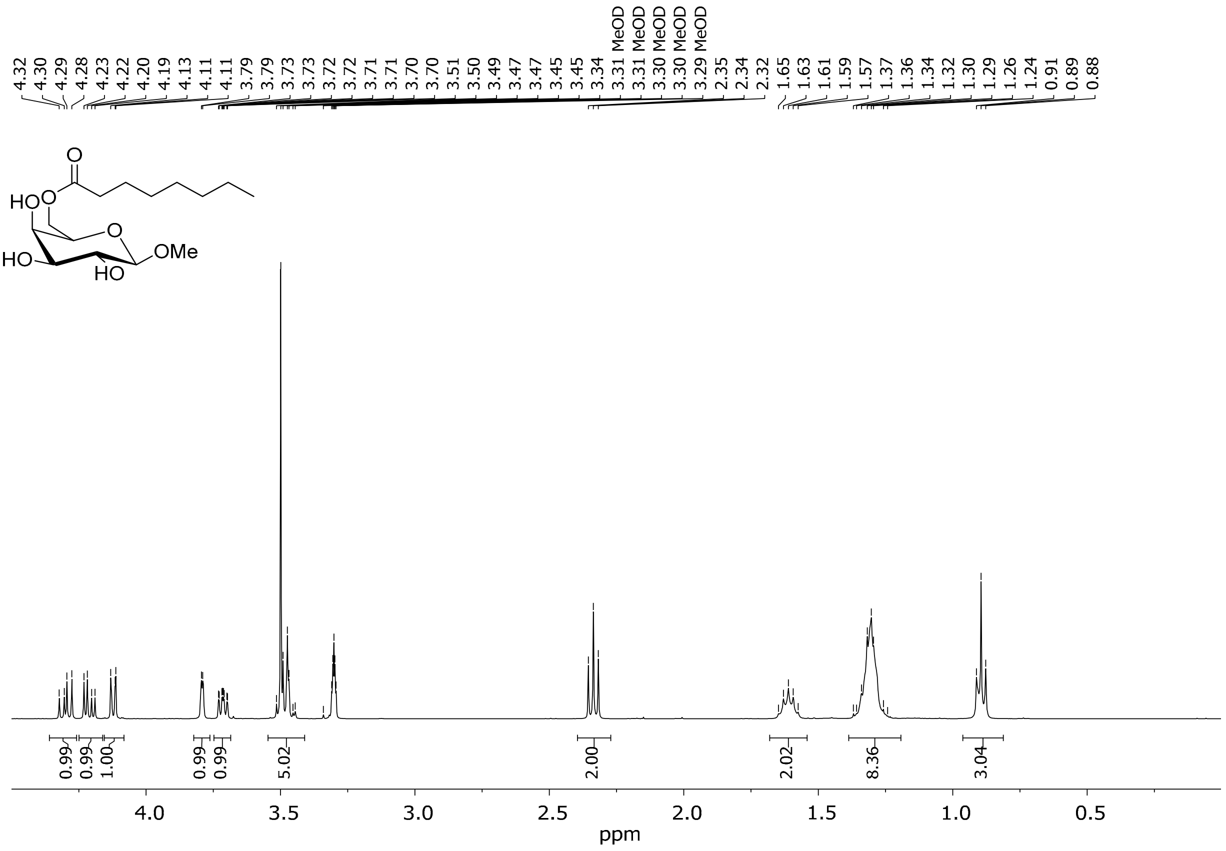

Figure S9. ^{1}H-NMR spectrum of methyl 6-*O*-octanoyl-β-D-galactopyranoside **12**.

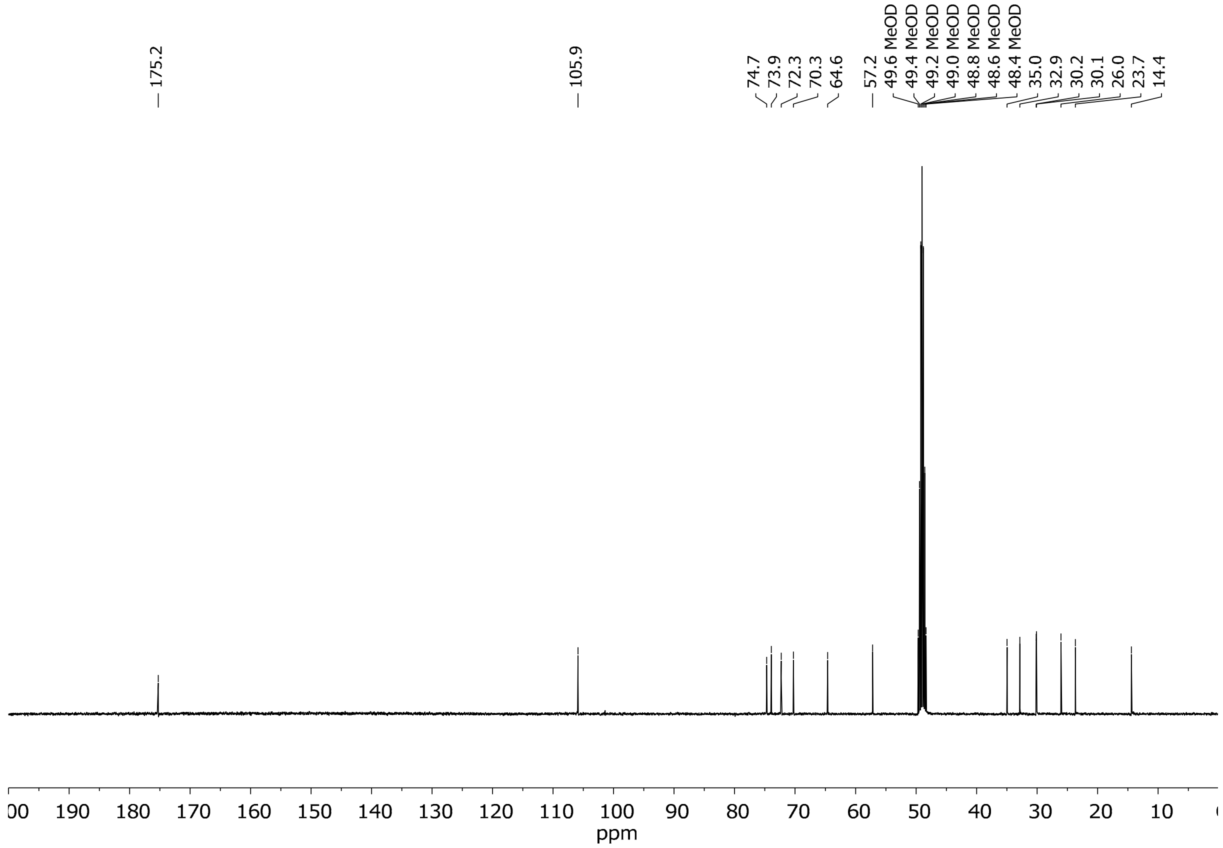

Figure S10. ^{13}C-NMR spectrum of methyl 6-*O*-octanoyl-β-D-galactopyranoside **12**.
